# Comparative transcriptional profiling analysis of olive ripe-fruit pericarp and abscission zone tissues shows expression differences and distinct patterns of transcriptional regulation

**DOI:** 10.1186/1471-2164-14-866

**Published:** 2013-12-09

**Authors:** Ruben Parra, Miguel A Paredes, Isabel M Sanchez-Calle, Maria C Gomez-Jimenez

**Affiliations:** Department of Plant Physiology, University of Extremadura, Avda de Elvas s/n, Badajoz, 06006 Spain; Department of Plant Physiology, University of Granada, Campus de Cartuja s/n, Granada, 18071 Spain

**Keywords:** Abscission, Gene expression, *Olea europaea*, 454 Pyrosequencing, Ripening, Transcriptomic comparative

## Abstract

**Background:**

In fleshy fruit, abscission of fully ripe fruit is a process intimately linked to the ripening process. In many fruit-tree species, such as olive (*Olea europaea* L. cv. Picual), there is a coupling of the full ripening and the activation of the abscission-zone (AZ). Although fully ripe fruit have marked physiological differences with respect to their AZs, dissimilarities in gene expression have not been thoroughly investigated. The present study examines the transcriptome of olive fruit and their AZ tissues at the last stage of ripening, monitored using mRNA-Seq.

**Results:**

Roche-454 massive parallel pyrosequencing enabled us to generate 397,457 high-quality EST sequences, among which 199,075 were from ripe-fruit pericarp and 198,382 from AZ tissues. We assembled these sequences into 19,062 contigs, grouped as 17,048 isotigs. Using the read amounts for each annotated isotig (from a total of 15,671), we identified 7,756 transcripts. A comparative analysis of the transcription profiles conducted in ripe-fruit pericarp and AZ evidenced that 4,391 genes were differentially expressed genes (DEGs) in fruit and AZ. Functional categorization of the DEGs revealed that AZ tissue has an apparently higher response to external stimuli than does that of ripe fruit, revealing a higher expression of auxin-signaling genes, as well as lignin catabolic and biosynthetic pathway, aromatic amino acid biosynthetic pathway, isoprenoid biosynthetic pathway, protein amino acid dephosphorylation, amino acid transport, and photosynthesis. By contrast, fruit-enriched transcripts are involved in ATP synthesis coupled proton transport, glycolysis, and cell-wall organization. Furthermore, over 150 transcripts encoding putative transcription-factors (TFs) were identified (37 fruit TFs and 113 AZ TFs), of which we randomly selected eight genes and we confirmed their expression patterns using quantitative RT-PCR.

**Conclusion:**

We generated a set of EST sequences from olive fruit at full ripening, and DEGs between two different olive tissues, ripe fruit and their AZ, were also identified. Regarding the cross-talk between fruit and AZ, using qRT-PCR, we confirmed a set of TF genes that were differentially expressed, revealing profiles of expression that have not previously been reported, this offering a promising beginning for studies on the different transcription regulation in such tissues.

**Electronic supplementary material:**

The online version of this article (doi:10.1186/1471-2164-14-866) contains supplementary material, which is available to authorized users.

## Background

Olive (*Olea europaea* L.), of worldwide economic importance, has high intra-specific genetic variation with a genome size of about 1,800 Mb [1]. This feature serves to analyze biological processes of biotechnological interest such as phenolic and lipid metabolism during fruit development [2–4] as well as terpenoids and sterols [5]. Directly or indirectly, these processes all affect the quality of olive oil as well as its nutritional profile. The genomic data on olive is augmenting through advances in mapping the olive genome [6, 7], and the DNA of the whole plastome of ‘Frantoio’, an Italian cultivar, has been sequenced [8]. Also, sequencing of the olive genome has been undertaken in Italy through the project OLEA (http://www.oleagenome.org/). Concomitantly, a number of large datasets of expressed sequence tag (EST) datasets have recently been reported for olive, generating 261,485 ESTs [2] and 443,811 ESTs [9] employing the 454 pyrosequencing technologies, an additional 1,132 ESTs with the use of suppression subtractive hybridization [3], as well as 2 million ESTs using Sanger and 454 pyrosequencing technologies [10], this being important for extending the catalog of olive transcripts in order to facilitate gene discovery, functional analysis, and molecular breeding.

Fruit ripening, abscission and senescence are key physiological events that occur during the growth and development of higher plants. These bear commercial implications both for the plant and the harvest. In agricultural research, the manipulation of genes governing these phenomena is key in order to develop varieties that can produce fruits with longer shelf lives as well as crops that tolerate greater environmental stress. Given that several genes are involved in these processes, the manipulation of complex traits such as ripening, abscission, and senescence is not feasible using single genes, and therefore efforts are being focused on specific transcription factors (TFs) that control entire pathways [11].The development of olive fruit involves complex processes following a double sigmoidal growth curve which lasts for 4-5 months and is influenced by numerous factors, including genotype [12, 13]. Olive-fruit properties at the time of harvest, including the final mix of primary and secondary metabolites that accumulate during ripening, largely determine the quality of the resulting oil and fruit. Recent transcriptomic and metabolic studies have demonstrated changes taking place during the development of the olive-fruit and the beginning of ripening [3, 4]. Progress in determining the transcriptome of the olive in terms of functional annotation and the assignment of gene ontology have made it possible to accurately describe of differences in gene expression between olive tissues [2, 3]. However, transcriptome information of the olive fruit at full ripening has not yet been determined.

After fruit ripening, many fruit-tree species undergo massive natural fruit abscission. In olive, abscission of mature fruit depends on the activation of the abscission zone (AZ) located between the pedicel and fruit, and the patterns of mature fruit abscission differ between cultivars [14, 15]. In some olive cultivars (cv. Picual), fruit ripening associated events lead finally to the abscission of the ripe fruit from the pedicel, this taking place at 217 days post-anthesis (DPA) [14, 15]. In a previous study, we reported the comparison of the Picual fruit AZ transcriptomes at two different stages (pre-abscission vs. abscission) using the RNA-Seq technique; 148 Mb of sequences (443,811 good-quality sequence reads) resulted and 4,728 differentially expressed genes were identified from these two samples [9]. Among the 70 TF genes induced during mature-fruit abscission in the olive AZ, the classes that are well represented included bZIP proteins, MYB proteins, and homeobox domain proteins [9]. The comparison between AZ and fruit allow us to restrict the set of genes putatively related to the abscission, and in this direction the results may hold worthwhile perspectives for the study of this process. Cross-talk between the two tissues may involve different components of the signaling network, such as TFs and other signaling molecules, playing either direct or indirect roles. However, molecular-genetic information on the relationship between ripe fruit and AZ is still very limited. In this study, using 454 pyrosequencing technology, we analyzed the overall transcriptional profile of olive (cv. Picual) fruit pericarp at full ripening to significantly expand the olive transcript catalog. We focused on comparing the transcriptomes generated from pericarp and AZ tissues of ripe fruit to establish the divergences as well as similarities in transcriptional networks, and especially to characterize the biological processes and transcriptional regulators enriched in gene clusters that are differentially regulated. Here, we found a total of 397,457 ESTs assembled into 17,048 isotigs, for which we made extensive annotations. In total, we identified 4,391 differentially expressed genes (DEGs) in ripe fruit and AZ, and characterized their biological functions using gene ontology (GO) annotation and KEGG pathway analysis. The results from this study show that distinct patterns of transcriptional regulation occurs among ripe fruit and their AZ in olive, identifying common and distinct TFs that have not been previously related to fruit ripening or abscission.

## Results and discussion

### 454 sequencing of olive transcriptomes

**Figure 1:**
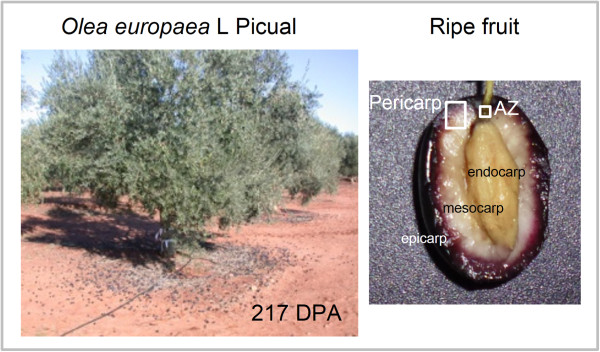
**Tissues of olive (** ***Olea europaea*** **L. Picual) at 217 DPA used in sequencing: pericarp (epicarp and mesocarp) of ripe fruit and AZ.** DPA: days post-anthesis.

To characterize olive transcriptomes and generate expression profiles between fruit ripening and abscission, Roche/454 GS-FLX (Titanium) pyrosequencing technology was used to sequence two cDNA samples from fruit pericarp and the AZ, which were collected from olive (cv. Picual) fruits at the ripe stage (217 DPA), when abscission occurs (Figure 1). After the cDNA libraries were prepared, their pyrosequencing was finished, and initial quality filtering was performed with the default parameters. The runs gave a total of 199,075 high-quality sequence reads for fruit pericarp, and 198,382 high-quality sequence reads for AZ (Additional file 1). Thus, a total of 397,457 high-quality ESTs were found for the two study samples. Additional file 2 offers a general view of the sequencing and assembly processes which provides the length distribution for these high-quality reads. Although many reads were very short (<100), over 80% were 300 to 500 bp in length. We assembled these sequences into 19,062 contigs (Additional file 2) grouped into 17,048 isotigs (7,003 for fruit, and 10,045 for AZ, respectively) (Additional file 1; Additional file 2). The average length of the contigs was around 500 bases and most of the contigs had fewer than 10 reads (Additional file 2). We assembled most of the high-quality reads (55%) into longer contigs, implying high coverage for these sequencing data. We then found over 10,000 UniProt identities using BLAST analysis on the sequences assembled (Additional file 1). Some 40% of the isotigs failed to map to UniProt identities, thus constituting a source to discover new genes.

### Comparison of olive transcriptomes between fruit and AZ tissues

**Figure 2:**
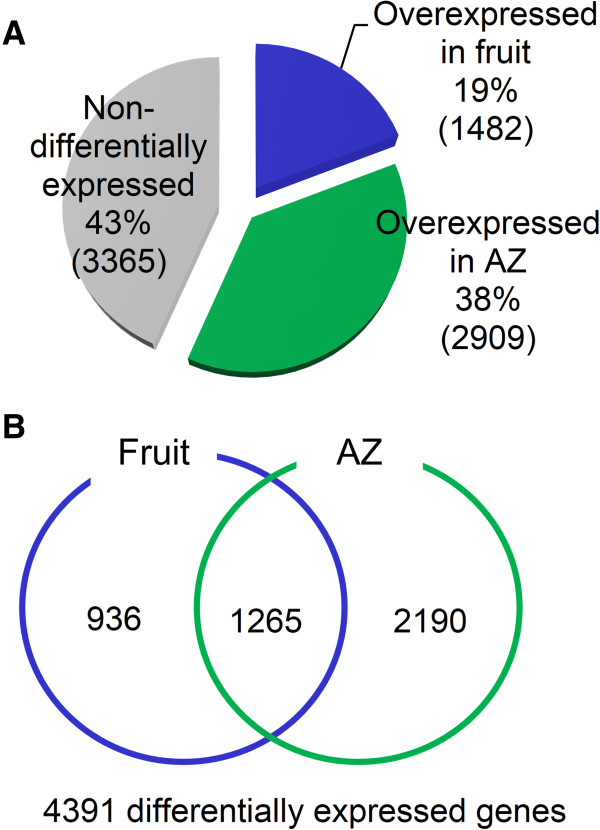
**Distribution of genes differentially expressed between olive ripe fruit and its AZ at 217 DPA. (A)** The number and percentages of overexpressed genes by tissue: olive fruit and AZ at late stage of ripening. **(B)** Overlap of overexpressed fruit genes and overexpressed AZ genes. This figure shows the number the transcripts that were specific for each tissue.

To investigate ripening-abscission distinctions, we compared the transcriptomes of olive fruit and AZ at full ripening (fruit-pericarp vs. fruit-AZ at 217 DPA). Read amounts for each of the 15,671 annotated isotigs (6,533 for fruit, and 9,138 for AZ) lead to the identification of 7,756 transcripts in our experiment (Additional file 3), which 4,391 were differentially expressed genes (DEGs); hereafter, these are called group I (P < 0.01), whereas the other genes (43%) having either low read abundance or non differential representation are called group II (Figure 2A). Thus, the comparative analysis of the transcription profiles conducted in pericarp and AZ of ripe fruit evidenced that a huge number of genes are differentially expressed in fruit and AZ. Of these 4,391 DEGs (Additional file 4), 1,482 showed a higher expression in the fruit pericarp, while 2,909 were overexpressed in the AZ at 217 DPA (Additional file 5; Additional file 6). A comparison of the DEGs indicated that 1,265 genes of these were common in both tissues, whereas 936 DEGs were expressed only in fruit (fruit genes), and 2,190 DEGs were expressed exclusively in AZ at 217 DPA (AZ genes) (Figure 2B). Thus, we identified a large number of fruit and AZ genes, implying that they participate in physiological processes exclusive to certain tissues.

**Figure 3:**
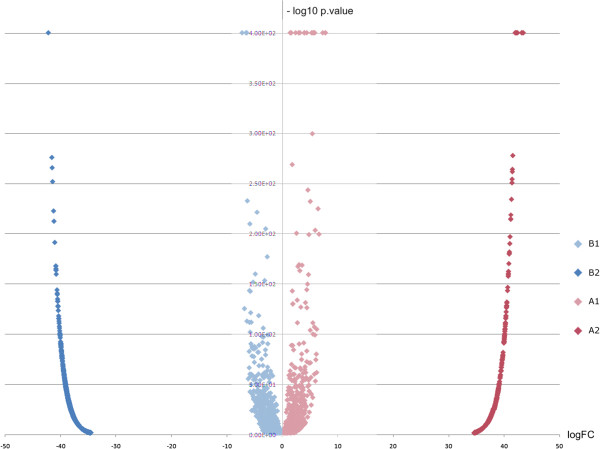
**Volcano blots show significant changes in gene expression between fruit and AZ tissues at 217 DPA.** Dispersion graph of the–log10 p value (y axis) against the logFC (x axis) corresponding to the genes clustered by their differential expression: A1 (fruit-enriched genes), A2 (fruit genes), B1 (AZ-enriched genes) and B2 (AZ genes). Fold changes and their associated *P* values for all probe sets can be found in Additional file 7.

To determine which cell processes might be critical in the last stage of fruit ripening in both tissues, we grouped transcripts by their expression signatures in both samples. For group I genes, hierarchical cluster analysis enabled us to identify 2 major clusters, called A and B. Cluster A had the 1,482 most abundant transcripts in fruit-pericarp at 217 DPA, while cluster B bore the 2,909 most abundant transcripts in fruit-AZ at 217 DPA. Subsequently, we split these two clusters into two subclusters, (A1, A2) and (B1, B2), respectively (Additional file 7). We present volcano plots for each hierarchal cluster group and identify gene with both high fold change and significance (Figure 3, Additional file 7). Subcluster A1 had 555 transcripts, which were more abundant in the fruit-pericarp sample with lower expression levels in the fruit-AZ sample at 217 DPA (“fruit-enriched genes”). Meanwhile, cluster A2 contained the 936 expressed transcripts exclusively in the fruit-pericarp sample at 217 DPA (“fruit genes”). In the fruit-AZ sample, cluster B1 had the 710 most abundant transcripts and lower expression levels in the fruit-pericarp sample at 217 DPA (“AZ-enriched genes”), whereas cluster B2 included the 2,190 exclusively expressed transcripts in the fruit-AZ sample at 217 DPA (“AZ genes”).

For each cluster, the most abundant transcripts appear in Table 1. For the fruit-enriched transcripts, the greatest differential expression was found for a transcript participating in abscisic acid (ABA) stress ripening (coding for an abscission stress ripening-like protein), and a transcript coding for β-glucosidase involved in carbohydrate metabolic process, suggesting that such ripening processes as cell-wall alterations occur in fruit-pericarp at the last stages of olive ripening. Also, a significantly higher expression in ripe fruit vs. AZ tissues was found for an *ACO1* (1-aminocyclopropane-1-carboxylic acid oxidase 1) and *ETR1* (ethylene receptor 1) involved in ethylene biosynthesis and perception, respectively, suggesting that ACO1 as well as ETR1 may be instrumental in balancing ethylene biosynthesis needs with ethylene signaling requirements to full ripening in olive-pericarp. Another transcript coding for thaumatin-like protein, which is developmentally regulated particularly in fruits during ripening, but is also induced in response to biotic or abiotic stress [16] revealed a fruit-enriched expression pattern. Also, tubulins beta chain revealed a fruit-enriched expression pattern, signifying that activation of vesicle trafficking involving these tubulins may take part in fruit-pericarp during fruit ripening. On the other hand, the genes that encode anthocyanidin synthase, 6,7-dimethyl-8-ribityllumazine synthase, and alpha-expansin 8 (*EXP8*) were the genes most highly expressed among those expressed exclusively in olive fruit compared to AZ (Table 1). A key component in the riboflavin pathway, 6,7-dimethyl-8-ribityllumazine synthase or CORONATINE INSENSITIVE1 SUPPRESSOR (COS1) is involved in jasmonic acid mediated signaling pathway [17]. This suggests that COS1 may participate in jasmonate signaling to regulate olive ripening, but not to regulate abscission of mature fruit. Previous works have shown that in many crops (e.g. grape [18], apple [19], litchi [20], and Chinese bayberry [21]) the anthocyanin content in fully ripe fruit correlates well with the cumulative expression of anthocyanin biosynthetic genes. In the present study, it was found that expression of anthocyanidin synthase was up-regulated in fruit-pericarp at full ripe stage, suggesting the regulation of anthocyanin biosynthesis by anthocyanidin synthase in the late olive-ripening stage. In addition, the strong up-regulation of *EXP8* indicates that this expansin plays a major role in cell-wall alterations involved in olive ripening.

**Table 1:** **The most abundant transcripts in fruit (Cluster A) and AZ (Cluster B) at the last stage of olive ripening**

Unigene ID	UniProt ID	Fruit	AZ	p-value	Description
**Cluster A**					
**Cluster A1**	**Enriched in fruit**				
OL006944	Q2TUW1	20742.30	1196.08	0.00E + 00	Abscisic stress ripening-like protein = *Glycine max*
OL007219	Q8GVD0	5033.88	214.76	0.00E + 00	Beta-glucosidase, Bglc = *Olea europaea* subsp. europaea
OL001156	B9H1F2	5022.65	95.79	0.00E + 00	Uncharacterized protein = *Populus trichocarpa*
OL006467	E0CU96	2920.45	169.91	0.00E + 00	Uncharacterized protein = *Vitis vinifera*
OL001418	B9R8J3	8902.90	1040.58	0.00E + 00	Phosphoprotein ECPP44 = *Ricinus communis*
OL007236	Q8H159	4247.26	1430.34	0.00E + 00	Polyubiquitin 10 [Cleaved into: Ubiquitin], UBQ10, At4g05320
OL003644	C6KMJ4	4794.95	1423.76	0.00E + 00	ACC oxidase, ACO1 = *Boea hygrometrica*
OL006886	Q0WLP3	5085.83	870.26	0.00E + 00	Uncharacterized protein = *Arabidopsis thaliana*
OL006727	P29512	1812.59	180.74	0.00E + 00	Tubulin beta-2/beta-3 chain, TUBB2 TUB2, At5g62690; TUBB3 TUB3, At5g62700
OL006856	Q06R56	1174.95	18.56	0.00E + 00	Acetyl-CoA carboxylase beta subunit accD, FEC0159 = *Forsythia europaea*
OL006553	O04111	1437.34	5.97	0.00E + 00	Chalcone synthase, CHS, *Perilla frutescens*
OL002387	B9S382	949.24	22.02	0.00E + 00	Tubulin beta chain = *Ricinus communis*
OL000027	A2IBF9	1329.71	22.64	0.00E + 00	Flavanone-3-hydroxylase = *Gossypium hirsutum*
OL002907	B9SLE5	3731.27	15.50	0.00E + 00	Peptidase = *Ricinus communis*
OL000014	A1E4D3	610.81	13.51	0.00E + 00	Ethylene receptor, ETR1 = *Coffea canephora*
OL003708	D5LY28	609.68	3.53	0.00E + 00	Soluble acid invertase 1, SAI1 = *Orobanche ramosa*
OL001944	B9RP00	1995.12	42.28	9.70E-301	Uncharacterized protein = *Ricinus communis*
OL007516	Q9LLB7	4587.02	1203.54	5.39E-270	Thaumatin-like protein = *Vitis vinifera*
OL001075	B7U8J4	1418.63	52.49	1.22E-244	Expansin, CDK3 = *Diospyros kaki*
OL005738	D7U0E8	538.58	14.77	2.73E-233	Uncharacterized protein = *Vitis vinifera*
OL007398	Q9AXU0	1854.70	19.23	5.46E-226	Major latex-like protein = *Prunus persica*
OL000584	A5BN70	585.33	8.71	2.72E-204	Uncharacterized protein = *Vitis vinifera*
OL006621	O49877	1057.22	162.37	2.53E-201	CYP1 (Cysteine protease TDI-65)
OL004008	D7SNI5	615.01	5.64	2.08E-200	Uncharacterized protein = *Vitis vinifera*
OL007235	Q8H145	432.58	14.77	5.11E-200	Putative elongation factor (Fragment), At1g56075
**Cluster A2**	**Fruit genes**				
OL003887	D7SKG3	9684.90	0.00	0.00E + 00	Uncharacterized protein = *Vitis vinifera*
OL004525	D7T2N4	5878.45	0.00	0.00E + 00	6,7-dimethyl-8-ribityllumazine synthase = *Vitis vinifera*
OL000028	A2ICC9	2823.47	0.00	0.00E + 00	Anthocyanidin synthase, ANS = *Vitis vinifera*
OL006333	E0CQN9	1291.93	0.00	0.00E + 00	Uncharacterized protein = *Vitis vinifera*
OL002413	B9S4E4	2111.11	0.00	0.00E + 00	Alpha-expansin 8 = *Ricinus communis*
OL004078	D7SQ46	928.57	0.00	0.00E + 00	Uncharacterized protein = *Vitis vinifera*
OL002282	B9S053	1262.49	0.00	0.00E + 00	ATP synthase alpha subunit mitochondrial = *Ricinus communis*
OL003892	D7SKJ8	1091.87	0.00	0.00E + 00	Uncharacterized protein = *Vitis vinifera*
OL004267	D7SVD2	1810.56	0.00	5.65E-279	Uncharacterized protein = *Vitis vinifera*
OL006945	Q2UYU6	619.52	0.00	3.12E-265	Flavonoid-3′-hydroxylase = *Vitis vinifera*
OL003801	D7SI22	693.27	0.00	7.63E-263	Uncharacterized protein = *Vitis vinifera*
OL005180	D7TJ49	903.07	0.00	2.23E-255	Uncharacterized protein = *Vitis vinifera*
OL007481	Q9FXL4	642.40	0.00	8.53E-252	Elicitor inducible beta-1,3-glucanase, NtEIG-E76 = *Nicotiana tabacum*
OL004529	D7T2X5	1244.84	0.00	2.22E-235	Uncharacterized protein = *Vitis vinifera*
OL004452	D7T0N0	444.07	0.00	1.16E-219	Uncharacterized protein = *Vitis vinifera*
OL001743	B9RI89	699.45	0.00	1.76E-215	Serine-threonine protein kinase = *Ricinus communis*
OL007506	Q9LIC2	398.86	0.00	3.50E-215	Multispanning membrane protein-like, At3g13772
OL005327	D7TN33	2085.55	0.00	7.29E-198	Uncharacterized protein = *Vitis vinifera*
OL004599	D7T4I1	790.51	0.00	5.34E-191	Uncharacterized protein = *Vitis vinifera*
OL007004	Q40168	880.38	0.00	1.21E-182	Floral homeotic protein AGAMOUS, TAG1 = *Solanum lycopersicum*
OL001261	B9I6M7	505.91	0.00	2.94E-181	Uncharacterized protein = *Populus trichocarpa*
OL007205	Q84V57	368.45	0.00	1.16E-180	Pectinesterase = *Nicotiana benthamiana*
OL006690	P14721	454.41	0.00	4.27E-171	Dihydroflavonol-4-reductase, DFRA = *Antirrhinum majus*
OL006603	O24329	532.05	0.00	2.50E-163	Putative uncharacterized protein = *Ricinus communis*
OL007050	Q45QI7	831.88	0.00	3.09E-161	Chalcone-flavonone isomerase, CHI = *Camellia sinensis*
**Cluster B**					
**Cluster B1**	**Enriched in AZ**				
OL007063	Q53U35	466.67	39974.19	0.00E + 00	Similar to pathogenesis-related protein, STH-2 = *Solanum lycopersicum*
OL004910	D7TBW7	92.31	12814.10	0.00E + 00	Uncharacterized protein = *Vitis vinifera*
OL000784	A5C4X8	19.48	1499.53	0.00E + 00	Uncharacterized protein = *Vitis vinifera*
OL005534	D7TTS3	9.40	677.78	7.69E-234	Uncharacterized protein = *Vitis vinifera*
OL001130	B9GQM0	24.67	528.28	1.89E-222	Glycosyltransferase, CAZy family GT8 = *Populus trichocarpa*
OL001048	B3Y023	6.44	337.01	1.24E-210	Arginine decarboxylase, PpADC, *Prunus persica*
OL001934	B9RNU7	144.97	1060.02	1.01E-205	Protein phosphatase 2c = *Ricinus communis*
OL007508	Q9LJU7	141.11	842.87	6.39E-178	Purple acid phosphatase 18, PAP18 PAP30, At3g20500
OL000621	A5BSF5	14.55	385.83	8.80E-161	Uncharacterized protein = *Vitis vinifera*
OL004617	D7T4X3	86.61	712.16	3.01E-154	Uncharacterized protein = *Vitis vinifera*
OL000020	A1X877	6.01	209.08	1.26E-152	NRC1 = *Solanum lycopersicum*
OL002350	B9S255	11.95	656.04	2.81E-144	Uncharacterized protein = *Ricinus communis*
OL002844	B9SJN1	4.75	235.99	1.57E-143	Transcription factor hy5 = *Ricinus communis*
OL000814	A5C762	3.65	376.25	2.47E-126	Uncharacterized protein = *Vitis vinifera*
OL003935	D7SLN3	4.44	211.35	3.03E-122	Uncharacterized protein = *Vitis vinifera*
OL003232	B9SWQ3	15.25	265.80	2.37E-119	Serine/threonine protein kinase = *Ricinus communis*
OL000971	A9PCV7	6.58	498.02	7,68E-114	Uncharacterized protein = *Populus trichocarpa*
OL004147	D7SS09	8.28	365.56	8,33E-113	Uncharacterized protein = *Vitis vinifera*
OL003339	B9T0K9	6.41	353.47	1.06E-112	Plasminogen activator inhibitor 1 RNA-binding protein, putative = *Ricinus communis*
OL007507	Q9LJI5	50.33	494.78	6.20E-107	V-type proton ATPase subunit d1, VHA-D1, At3g28710
OL000585	A5BN72	2.24	113.57	6.46E-103	Uncharacterized protein = *Vitis vinifera*
OL001014	B1PK08	114.23	616.10	3.23E-100	Putative polygalacturonase = *Olea europaea*
OL007154	Q6RYA0	51.28	584.61	1,32E-98	Salicylic acid-binding protein 2 = *Nicotiana tabacum*
OL003709	D5M8I6	22.52	216.21	3.46E-98	Uncharacterized protein = *Vitis vinifera*
OL000614	A5BR22	108.46	1156.08	1.18E-92	Uncharacterized protein = *Vitis vinifera*
**Cluster B2**	**AZ genes**				
OL007111	Q68V46	0.00	1349.85	0.00E + 00	Beta-1,3-glucanase, glu-4 = *Olea europaea*
OL001027	B2M153	0.00	517.74	4.51E-277	Putative laccase = *Rosa* hybrid cultivar
OL002714	B9SF95	0.00	614.34	7.75E-267	Nitrate transporter = *Ricinus communis*
OL006675	O98664	0.00	576.60	5.46E-253	Ribulose bisphosphate carboxylase large chain, rbcL = *Kigelia africana*
OL007711	Q9XEL8	0.00	396.80	8.65E-224	3-hydroxy-3-methylglutaryl-coenzyme A reductase 2, HMGR2 = *Capsicum annuum*
OL000602	A5BPW9	0.00	602.46	2.97E-213	Uncharacterized protein = *Vitis vinifera*
OL001338	B9NAX4	0.00	3264.55	3.51E-192	Uncharacterized protein = *Populus trichocarpa*
OL000148	A5AN11	0.00	396.34	1.06E-168	Uncharacterized protein = *Vitis vinifera*
OL004086	D7SQA7	0.00	273.23	1.06E-168	Uncharacterized protein = *Vitis vinifera*
OL003142	B9STR3	0.00	277.60	5.43E-166	Endosomal P24A protein = *Ricinus communis*
OL002860	B9SK95	0.00	489.81	8.68E-165	12-oxophytodienoate reductase opr = *Ricinus communis*
OL005126	D7THY5	0.00	222.22	1.74E-164	Uncharacterized protein = *Vitis vinifera*
OL007151	Q6RH27	0.00	570.32	1.42E-160	NAC domain protein, SlNAC1 = *Solanum lycopersicum*
OL004686	D7T6Y2	0.00	305.72	6.41E-145	Uncharacterized protein = *Vitis vinifera*
OL007397	Q9AXR6	0.00	248.90	1.31E-141	ATP:citrate lyase = *Capsicum annuum*
OL000367	A5B7F7	0.00	319.83	2.10E-140	Uncharacterized protein = *Vitis vinifera*
OL002951	B9SML0	0.00	214.99	6.88E-136	Lyase = *Ricinus communis*
OL007180	Q7XE16	0.00	176.98	8.81E-134	Cell division cycle protein 48 = *Oryza sativa* subsp. japonica
OL005047	D7TFE6	0.00	357.56	1.76E-133	Uncharacterized protein = *Vitis vinifera*
OL000444	A5BDC8	0.00	382.41	5.77E-129	Uncharacterized protein = *Vitis vinifera*
OL002899	B9SL31	0.00	245.52	2.31E-128	Transcription factor = *Ricinus communis*
OL003084	B9SRT5	0.00	96.17	1.89E-124	Phospholipid-transporting atpase = *Ricinus communis*
OL007255	Q8LAH7	0.00	341.40	4.96E-119	12-oxophytodienoate reductase 1, AtOPR1, At1g76680
OL001800	B9RJM7	0.00	394.51	1.27E-116	Uncharacterized protein = *Ricinus communis*
OL002929	B9SM03	0.00	296.74	2.54E-116	Uncharacterized protein = *Ricinus communis*

The sequences were selected at p < 0.01 and were sorted by p-value. The table shows the total read count in RPKMx1000 for each gene after normalization across the 2 samples: (a) Fruit at 217 DPA, (b) AZ at 217 DPA.

Among the most abundant AZ-enriched transcripts, we identified a homolog of *STH-2* (Similar to pathogenesis-related protein 2) (Table 1), encoding a pathogenesis-related protein (PR), which are observed in the olive AZ during the induction of mature-fruit abscission [9]. However, further work is necessary to ascertain the biological significance of pathogenesis-related gene expression in the olive AZ during abscission. In pea, there is an accumulation of *STH2* homologs during late embryogenesis [22], and in *Craterostigma plantagineum* during rehydration of desiccated plants [23]. In addition, a homolog of *PAP18* (At3g20500), encoding a purple acid phosphatase (PAP) induced to phosphate limitation [24], and a homolog of glutamine synthetase, were very significantly expressed in fruit-AZ compared to fruit-pericarp tissue, indicating a role for these proteins in intercellular transport during mature-fruit abscission. PAPs, metallophosphoesterases that contain a bimetal nucleus in their active center [25], were involved in plant tolerance to phosphate limitation [24]. Previous experiments showed that, in phloem companion cells, glutamine synthetase activity affects proline levels [26]. The predominant expression of glutamine synthetase suggests redistribution of proline within the AZ during abscission. Among the most abundant AZ genes (Table 1, Cluster B2), cell wall-related genes were detected. This was expected because the main changes in texture related to cell separation result from enzyme-mediated structural and compositional changes in the cell wall. This includes, for example, a beta-1,3-glucanase, which catalyze the hydrolysis of β-1,3-glucan linkages of callose, as well as participating in many processes including cell-wall remodeling, secondary-wall formation, and phytohormone activation [27]. Reportedly, abscission induction is accompanied by the marked up-regulation of a gene that encodes β-1,3-glucanase, as well as the down-regulation of a gene that encodes a callose synthase in the fruit-AZ [9]. This activation of beta-1,3-glucanase was stronger in olive AZ, showing that this phenomenon is related to fruit abscission in olive. Also, one gene associated with nitrate transport is among AZ genes, suggesting the function of nitrate as an important ion for fruit abscission.

### Gene ontology functional enrichment analysis of differentially expressed genes

**Figure 4:**
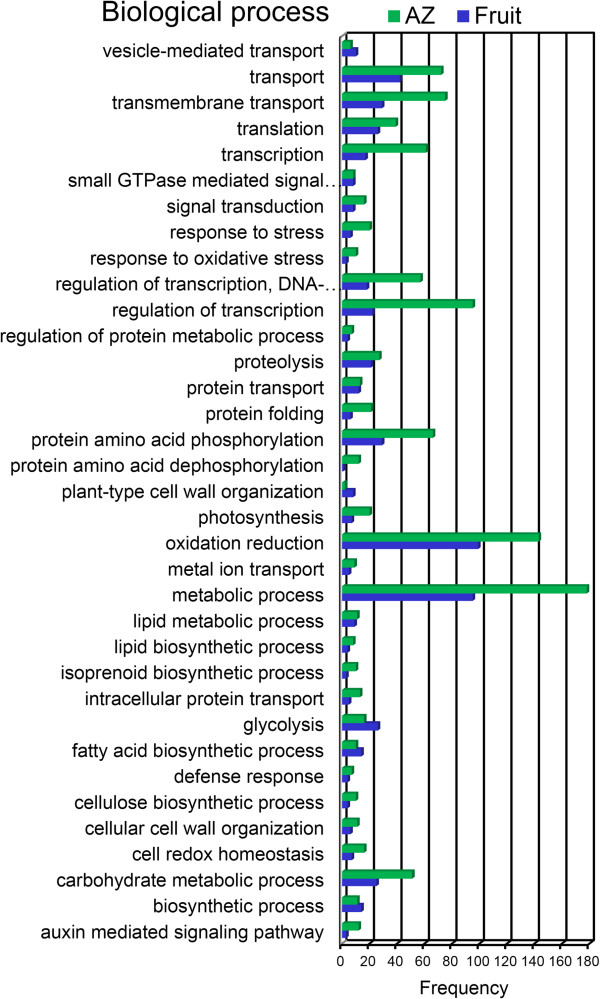
**Comparison of GO “biological process” term frequencies in overexpressed unigenes.** Comparison of the occurrence frequencies of the GO “biological process” terms in the GO annotations of the unigenes of the 1,491 overexpressed unigenes in olive fruit and the 2,900 overexpressed transcripts in olive AZ at 217 DPA. The number of occurrences is given for the most frequent terms.

**Figure 5:**
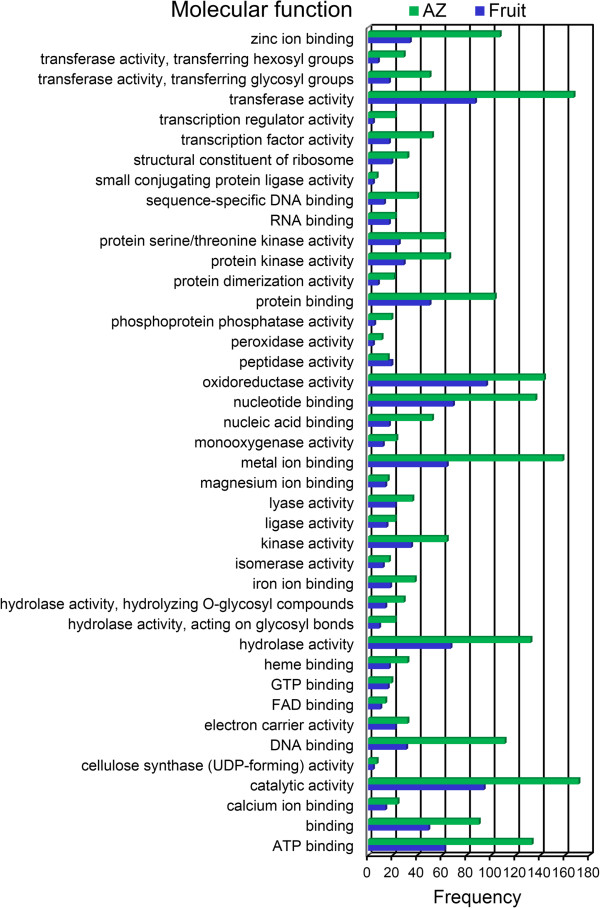
**Comparison of GO “molecular function” term frequencies in overexpressed unigenes.** Comparison of the occurrence frequencies of the GO “molecular function” terms in the GO annotations of the unigenes of the 1,491 overexpressed unigenes in olive fruit and the 2,900 overexpressed transcripts in olive AZ at 217 DPA. The number of occurrences is given for the most frequent terms.

**Figure 6:**
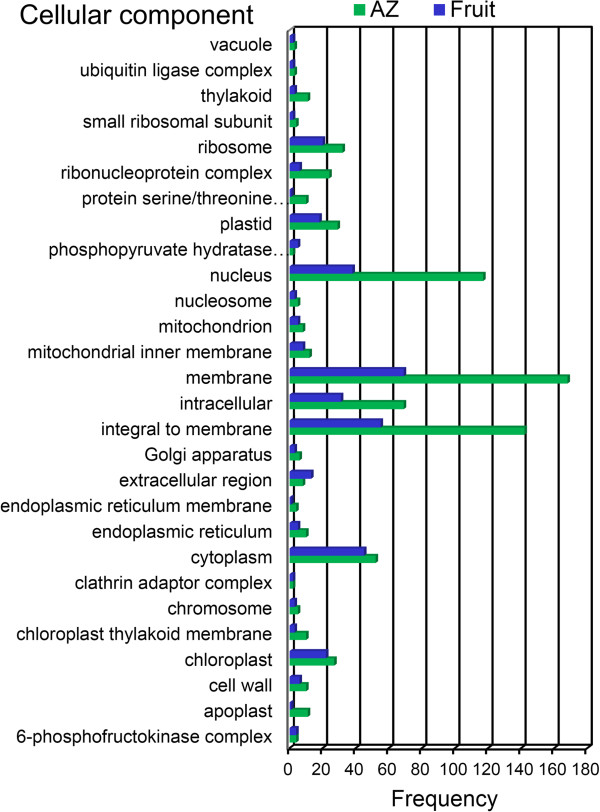
**Comparison of GO “cellular component” term frequencies in overexpressed unigenes.** Comparison of the occurrence frequencies of the GO “cellular component” terms in the GO annotations of the unigenes of the 1,491 overexpressed unigenes in olive fruit and the 2,900 overexpressed transcripts in olive AZ at 217 DPA. The number of occurrences is given for the most frequent terms.

To provide a general view on the functions and processes that change in fruit and AZ at the last stage of ripening, we classified the differentially expressed genes using the Gene Ontology (GO) database. In addition, based on their sequence similarities, we assigned GO accessions to the differentially expressed genes to identify the proteins in the UniProt database annotated with GO accessions in addition to the InterPro and Pfam domains they contained. Among the 15,671 annotated isotigs, 7,433 were designated at least one GO term (Additional file 1, Additional file 8). The GO terms “Oxidation reduction”, “Oxidoreductase activity”, and “Membrane” were the most represented ones among the biological process (Figure 4), molecular function (Figure 5), and cellular component categories (Figure 6), respectively.

Also GO terms were identified in the category of biological processes that proved to be over-represented in the lists of genes that showed higher expression in ripe fruit and AZ tissues, respectively (Figure 4). These GO terms constitute indicators of different biological processes that two different tissues underwent in the last stage of ripening. A number of GO classifications proved to be over-represented in genes which had augmented transcript accumulation in fruit at the last stage of ripening. The over-represented group in fruit at 217 DPA having the greatest number among the differentially expressed genes was “Oxidation reduction”, “Metabolic process”, “Transport”, “Transmembrane transport”, “Protein amino acid phosphorylation”, “Glycolysis” and “Carbohydrate metabolic process” (Figure 4). Remarkably, the AZ at 217 DPA also bore a significant representation of transcripts associated with “Metabolic process”, “Oxidation reduction”, “Regulation of transcription”, “Transmembrane transport”, “Transport”, and “Protein amino acid phosphorylation” (Figure 4). Thus, GO terms including “Oxidation reduction”, “Transport”, “Transmembrane transport”, “Protein amino acid phosphorylation”, and “Carbohydrate metabolic process”, were enriched in both lists of genes (Figure 4), indicating that the same biological processes might necessitate different gene sets in two different tissues during full ripening and abscission to support their activities. Sharp differences nevertheless appeared between the two lists of enriched GO terms. Notably, GO terms associated with aromatic amino acid family biosynthetic process, lignin catabolic and biosynthetic process, isoprenoid biosynthetic process, protein amino acid dephosphorylation, amino acid transport, photosynthesis, auxin signaling pathway, apoptosis, defense responses, and responses to stresses were highly enriched in genes more highly expressed in the olive AZ, while differences with respect to other enriched GO terms included ATP synthesis coupled proton transport, glycolysis, and plant-type cell-wall organization which underwent enrichment in genes of higher expression in ripe fruits, suggesting that such biological processes may be associated with ripening-abscission distinctions.

The profile of abundant transcripts in olive ripe fruit (217 DPA) indicates a predominant expression of proteins related to “Oxidoreductase activity”, “Catalytic activity”, “Transferase activity”, “Hydrolase activity”, as well as, “Nucleotide binding”, “Metal-ion binding”, and “ATP binding”, while the “Catalytic activity”, “Transferase activity”, and “Metal-ion binding” GO term was the most over-represented term for the genes in the olive AZ at 217 DPA (Figure 5). Differences of other enriched GO terms included 2-alkenal reductase activity, acyltransferase activity, amino acid transmembrane transporter activity, antiporter activity, drug transmembrane transporter activity, phosphoprotein phosphatase activity, ATP binding, calcium-ion binding, DNA binding, heme binding, and zinc-ion binding which proved to be enriched in genes that showed higher expression in AZ, while acetyl-CoA carboxylase activity, cysteine-type endopeptidase activity, and hydrogen ion transmembrane transporter activity, which were found to be enriched in genes more abundantly expressed in ripe fruit.

Finally, within the “Cellular compartment” category, the “Membrane”, “Integral to membrane” and “Cytoplasm” GO terms constituted the most overrepresented category for the genes with increased transcript accumulation in ripe fruit at 217 DPA (Figure 6). The distribution of gene functions (according to GO assignment) in the fruit and the AZ transcriptomes were largely similar, especially in the categories of molecular function and metabolism, but also different gene functions. These annotations constitute a useful resource for research on gene function, cellular structures, and processes in the two tissues studied.

### Metabolic pathways in the last stage of fruit ripening

The olive transcriptomes at the last stage of fruit ripening from our experiment provide the means to examine metabolic and other pathways which differ between the two tissues during this process. GO enrichment identified metabolic pathways that may be key to the last stage of fruit ripening and abscission. To delineate these metabolic pathways further, we mapped the Kyoto Encyclopedia of Genes and Genomes (KEGG; http://www.genome.jp/kegg) [28] database to the annotations in our transcript data. Of the 10,139 detected proteins in our experiment, 1,442 were annotated with 1,034 Enzyme Commission (EC) codes and mapped to 137 different KEGG pathways (Additional file 9).

**Figure 7:**
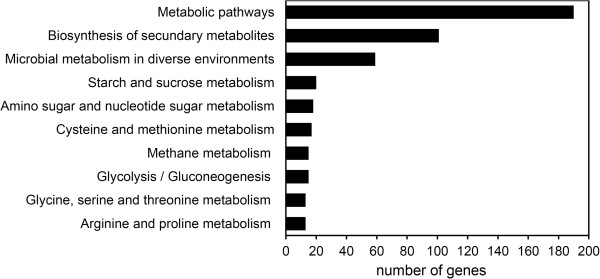
**Histogram illustrating pathway enrichment analyses.** Distribution of the number of differentially expressed genes between ripe fruit and AZ tissues in different metabolic pathways.

GO term representation of all differentially expressed genes between fruit and AZ tissues at 217 DPA is shown in Figure 7. This revealed significantly enriched pathways: biosynthesis of secondary metabolites (101 enzymes represented), microbial metabolism in diverse environments (59), starch and sucrose metabolism (20, Additional file 10, Table 2), amino sugar and nucleotide sugar metabolism (18, Additional file 11, Table 2), cysteine and methionine metabolism (17, Additional file 12, Table 2), methane metabolism (15, Additional file 13), glycolysis/gluconeogenesis (15, Additional file 14), glycine, serine, and threonine metabolism (13, Additional file 15), and arginine and proline metabolism (13, Additional file 16).

**Table 2:** **List of olive transcripts from KEGG pathway maps with EC numbers, Unigene ID numbers, UniProt ID numbers, normalized expression values and annotated gene description**

KEGG PATHWAY	EC	Unigene ID	UniProt ID	Fruit	AZ	p-value	Description
**STARCH AND SUCROSE METABOLISM**							
**Cluster A**							
**Cluster A1**	**Enriched in fruit**						
	3.2.1.26	OL003708	D5LY28	610	4	0.00E + 00	Beta-fructofuranosidase
	2.4.1.13	OL000884	A7IZK5	244	85	1.61E-36	Sucrose synthase
	3.2.1.39	OL002642	B9SCU1	111	94	5.10E-22	Glucan endo-1,3-beta-D-glucosidase
**Cluster A2**	**Fruit genes**						
	2.4.1.1	OL002542	B9S939	250	0	3.20E-96	1,4-alpha-glucan phosphorylase
	2.4.1.12	OL002104	B9RUD8	5	0	3.91E-03	Cellulose synthase
	2.7.7.27	OL000035	A3KCF8	10	0	6.10E-05	Glucose-1-phosphate adenylyltransferase
	2.7.7.9	OL002654	B9SD97	5	0	1.95E-03	UDP glucose pyrophosphorylase
	3.1.1.11	OL001166	B9H3W4	10	0	3.91E-03	Pectin methylesterase
	5.1.3.6	OL007529	Q9LPC1	8	0	9.77E-04	UDP-glucuronate 4-epimerase
	5.3.1.9	OL003650	C6TGC6	6	0	1.95E-03	Glucose-6-phosphate isomerase
**Cluster B**							
**Cluster B1**	**Enriched in AZ**						
	3.2.1.2	OL006254	E0AE02	18	32	1.82E-03	Beta-amylase
	4.1.1.35	OL001047	B3VDY9	102	168	2.19E-06	UDP-glucuronate decarboxylase
	2.4.1.21	OL001761	B9RIR1	5	28	3.24E-08	ADP-glucose synthase
**Cluster B2**	**AZ genes**						
	3.2.1.4	OL007034	Q43149	0	20	9.31E-10	Cellulase
	2.7.1.4	OL006635	O65583	0	9	2.44E-04	Fructokinase
	3.1.3.12	OL002988	B9SNT9	0	4	1.95E-03	Trehalose 6-phosphate phosphatase
	3.2.1.1	OL007022	Q42678	0	17	2.38E-07	Alpha-amylase
	3.2.1.15	OL000895	A7PZL3	0	10	3.05E-05	Polygalacturonase
	3.2.1.20	OL007491	Q9LEC9	0	12	2.91E-11	Alpha-glucosidase
**CYSTEINE AND METHIONINE METABOLISM**							
**Cluster A**							
**Cluster A1**	**Enriched in fruit**						
	2.6.1.1	OL001190	B9HAW0	35	8	3.18E-06	Aspartate transaminase
	2.7.1.100	OL002217	B9RY82	64	24	1.36E-05	S-methyl-5-thioribose kinase
**Cluster A2**	**Fruit genes**						
	2.1.1.14	OL002466	B9S6C1	81	0	1.29E-26	5methyltetrahydropteroyltriglutamate-homocysteine S-methyltransferase
	2.1.1.37	OL001007	B0FPD7	6	0	1.95E-03	DNA (cytosine-5-)-methyltransferase
	2.5.1.47	OL002734	B9SFU8	52	0	4.34E-19	Cysteine synthase
	2.5.1.6	OL007215	Q8GTL5	10	0	2.44E-04	Methionine adenosyltransferase
**Cluster B**							
**Cluster B1**	**Enriched in AZ**						
	2.5.1.48	OL002235	B9RYU1	3	23	1.94E-06	Cystathionine gamma-synthase
	2.8.1.2	OL001730	B9RHZ9	7	57	2.12E-13	3-mercaptopyruvate sulfurtransferase
	3.1.3.77	OL006405	E0CSI1	5	17	5.08E-04	Acireductone synthase
	3.3.1.1	OL006738	P35007	239	308	2.02E-06	Adenosylhomocysteinase
	5.3.1.23	OL002002	B9RR88	12	74	7.52E-15	S-methyl-5-thioribose-1-phosphate isomerase
**Cluster B2**	**AZ genes**						
	1.1.1.27	OL007383	Q96569	0	191	9.54E-07	L-lactate dehydrogenase
	1.14.17.4	OL006733	P31237	0	67	1.36E-20	ACC oxidase
	2.3.1.30	OL006733	B9S9Q4	0	27	1.16E-10	Serine O-acetyltransferase
	2.5.1.16	OL003685	D2K8S6	0	52	5.55E-17	Spermidine synthase
	4.1.1.50	OL000082	A5AFT0	0	133	2.80E-45	Adenosylmethionine decarboxylase
	2.7.2.4	OL005688	D7TYU1	0	5	7.81E-03	Aspartate kinase
**AMINO SUGAR AND NUCLEOTIDE SUGAR METABOLISM**							
**Cluster A**							
**Cluster A1**	**Enriched in fruit**						
	5.3.1.8	OL001163	B9H303	77	5	1.21E-21	Mannose-6-phosphate isomerase
	5.3.1.9	OL001141	B9GV29	16	4	7.53E-04	Glucose-6-phosphate isomerase
**Cluster A2**	**Fruit genes**						
	1.1.1.271	OL002645	B9SCY0	17	0	3.05E-05	GDP-L-fucose synthase
	2.7.1.4	OL004560	D7T3P0	12	0	6.10E-05	Fructokinase
	2.7.7.27	OL001390	B9R7X6	7	0	9.77E-04	Glucose-1-phosphate adenylyltransferase
	2.7.7.64	OL007403	Q9C5I1	11	0	1.91E-06	UTP-monosaccharide-1-phosphate uridylyltransferase
	2.7.7.9	OL002654	B9SD97	5	0	1.95E-03	UTP-glucose-1-phosphate uridylyltransferase
	5.1.3.6	OL007529	Q9LPC1	8	0	9.77E-04	UDP-glucuronate 4-epimerase
**Cluster B**							
**Cluster B1**	**Enriched in AZ**						
	3.2.1.14	OL007471	Q9FS45	10	134	5.46E-30	Chitinase
	4.1.1.35	OL001047	B3VDY9	102	168	2.19E-06	UDP-glucuronate decarboxylase
**Cluster B2**	**AZ genes**						
	3.2.1.55	OL002630	B9SCF3	0	6	2.44E-04	Alpha-N-arabinofuranosidase
	5.1.3.12	OL002629	B9SQF3	0	6	3.91E-03	UDP-glucuronate 5”-epimerase
	5.4.2.8	OL003424	B9T3D2	0	5	9.77E-04	Phosphomannomutase

The table shows the total read count in RPKMx1000 for each gene after normalization across the 2 samples: (a) Fruit at 217 DPA, (b) AZ at 217 DPA. We selected the sequences at p < 0.01.

### Transcription factors in olive fruit at the late stage of ripening

**Figure 8:**
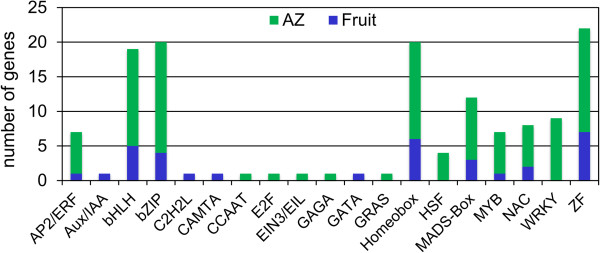
**Differentially expressed TF genes and classification of TF families.** Comparison of significantly overexpressed TF transcripts between fruit (blue) and AZ (green) at 217 DPA. Number of transcripts related to TFs in each TF family. The 150 TF genes were classified into 20 TF families.

**Figure 9:**
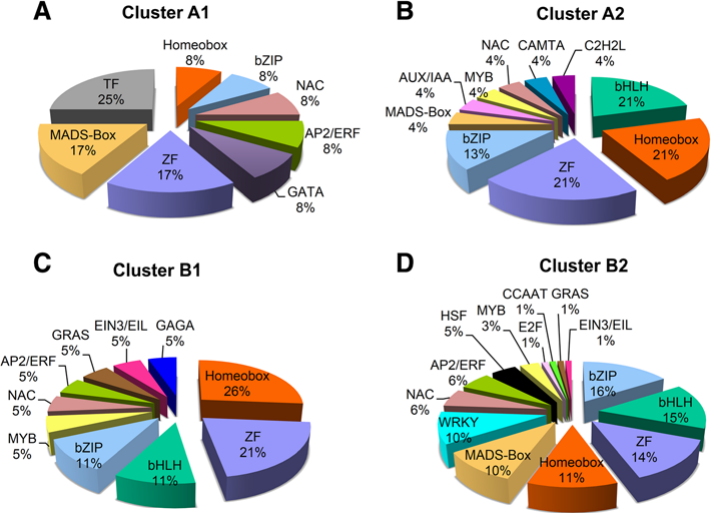
**Distribution of olive TF genes expressed at 217 DPA in fruit or AZ tissues, based on their family membership**. Fruit-(Cluster A1) or AZ-enriched (Cluster B1) and fruit (Cluster A2) or AZ (Cluster B2) TFs at the last stage of olive fruit ripening. **(A)** Fruit-enriched (Cluster A1) or **(B)** fruit (Cluster A2) TFs and **(C)** AZ-enriched (Cluster B1) or **(D)** AZ (Cluster B2) TFs at the last stage of olive fruit ripening.

Of 4,391 differentially expressed genes, 150 genes putatively encoding TF of diverse families were differentially expressed in olive AZ compared to fruit at 217 DPA (P < 0.01). The majority of these were induced in AZ (Figure 8, Additional file 17). Overall, 37 genes had peak read amounts within cluster A (the set of fruit-induced genes), and 113 genes within cluster B (the set of AZ-induced genes). Within cluster A, the most abundant TFs proved to be a MADS-box domain protein (AG1) detected within subcluster A2 (Additional file 17). Indeed, MADS-box proteins were the most abundant TFs in ripe fruit, two in subcluster A1 (TAGL2 and AGL9) and one in subcluster A2 (AG1), implying coordinated regulation of this class of TFs in ripe fruit (217 DPA). However, in cluster A the well-represented classes included homeobox domain proteins, zinc finger (ZF) proteins, basic helix-loop-helix (bHLH) proteins, and Basic Leucine Zipper (bZIP) proteins. Cluster A1 is enriched in the MADS-box and ZF TF families (Figure 9A, Additional file 17), whereas cluster A2 was rich in the bHLH, homeobox, ZF and bZIP families (Figure 9B, Additional file 17). The control of fleshy-fruit ripening involves many different TFs. In climacteric as well as non-climacteric fruits, a number of MADS-box genes reportedly regulate fruit development and ripening [29]. Master regulators in tomato are *HB-box* (*LeHB-1*), *MADS-box* (*SEP4-like*, *RIN*, *TDR4*, *TAG1*, *TAGL1*), *SBP-box* (*CNR*), and *NAC* genes [30]. A series of TFs, homologous to several of these master regulators, appear in ripe olive fruit (Additional file 17).

Similarly, the well represented classes in AZ tissue at the late stage of ripening (Cluster B) included ZF proteins, homeobox domain proteins, bHLH proteins, and bZIP proteins (Figure 8). Cluster B1 is enriched in ZF proteins and homeobox domain proteins (Figure 9C), whereas cluster B2 was found to be rich in the bHLH and bZIP families (Figure 9D). Thus, although two clusters containing members from several TF families, in each cluster, clearly significant difference was found in the proportion of families. Moreover, there are distinct TF families in each cluster: the Aux/IAA, C2H2L, CAMTA families in cluster A, and the HSF, GRAS, GAGA-binding protein, EIN3/EIL, E2F/DP, CCAAT-binding protein and WRKY families in cluster B (Figure 9). The enrichment of sequence elements in different gene groups from each cluster in combination with data on transcript abundance offer a tenable set of TFs which could bind these elements and that could be examined in future research.

Among the AZ-overexpressed TF types, HSF proteins, GRAS proteins, GAGA-binding protein, E2F/DP protein, and WRKY proteins were abundantly represented in the olive AZ during mature-fruit abscission [9]. The diversification and functional interaction of HSFs is known, as is their integration into the complex stress signaling and response networks of plants [31], and, a HSF-like TF, TBF1, have been identified as a key molecular mechanism for plant growth-to-defense transition [32]. In our analysis, 4 HSF TFs were exclusively overexpressed in olive-AZ (Additional file 17), supporting the idea that an increase of these HSF genes might be associated with mature-fruit abscission in olive AZ. Transcriptional regulators belonging to the GRAS family have been related to plant growth and development, as well as to biotic and abiotic stress [33]. Also, we report that several GRAS TFs, including homologs of *GRA1*, *GRAS4*, *GRAS6*, and *GRAS10* (*Solanum lycopersicum*), are exclusively overexpressed in the olive AZ (Additional file 17), suggesting that these GRAS TFs probably mediate abscission-responsive transcription. Ever since GAGA-binding proteins were identified and characterized in plants, few advances have been made in explaining their function. Another up-regulated gene in olive-AZ was a homolog of *BBR/BPC1* (*Vitis vinifera*), a GAGA-binding transcriptional activator (Additional file 17), indicating that this family control transcriptional activation of homeotic genes, probably started by ethylene, which potentially leads to the activation of abscission-related proteins in the olive AZ. E2F/DP family of TFs having critical and antagonistic functions in pathways involved in DNA repair, cell division, and differentiation. In olive, *E2F3*, encoding a key component of the cyclin D/retinoblastoma/E2F pathway that is a potent activator of E2F-responsive genes in Arabidopsis [34], was highly expressed during mature-fruit abscission in the AZ [9]. Here, we also identified one member of E2F family exclusively overexpressed in the AZ (Additional file 17). WRKY proteins are known to have a key part in plant defense against several types of biotic stress, developmental processes, and certain signal-transduction processes that are plant-hormone mediated (e.g. GA, ABA, or SA) [35]. Notably, our analyses have revealed that 9 *WRKY* genes (Additional file 17) are exclusively over-regulated in the olive AZ, which it is consistent with previous studies where the expression of some *WRKY* genes are induced during floral abscission [36] and mature-fruit abscission [9]. Thus, our data corroborate that, in the olive AZ, TFs belonging to these families may potentially help trigger the transcriptional cascade. Further study would be needed to reveal the molecular basis of gene expressional regulation.

Among the 37 TF genes induced in ripe fruit (Cluster A), 25 were exclusively expressed in fruit (Cluster A2, Additional file 17). We found it useful to consider these “fruit TFs” (Figure 9B) separately from 12 “fruit-enriched” TFs (Figure 9A), which were upregulated in ripe fruit compared to AZ at 217 DPA. The 25 genes encode 6 ZF proteins, 5 homeobox proteins, 5 bHLD domain class TFs, 3 bZIP, one MADS-box TF (AG1), one MYB TF (MYBA22), one NAC TF, one Aux/IAA (IAA1) protein, one CAMTA TF, and one C2H2L TF (Figure 9B, Additional file 17). This finding suggests that TFs from these families have potentially important roles in mediating late events during olive ripening. Similarly, among the 113 TF genes induced in the AZ at 217 DPA (Cluster B, Additional file 17), most of them (94) were exclusively expressed in the AZ compared to the ripe fruit (AZ TFs, cluster B2). These genes encoding 14 bZIP family TFs, 12 bHLH family TFs, 12 ZF proteins, 9 MADS-box family TFs, 9 homeobox family TFs, 9 WRKY family TFs, 5 NAC family TFs, 5 AP2/ERF family TFs, 5 MYB family TFs, 4 Heat shock factor (HSF) proteins, 3 GRAS proteins, one EIN3/EIL protein, one E2F protein and one CCAAT protein, among others (Figure 9D). The 10 most differentially overexpressed genes in the olive AZ encoding TFs were MYBPA1 (*Vitis vinifera),* one WRKY (*Ricinus communis),* MYB108-like protein 1 (*Vitis vinifera),* one ZF (*Ricinus communis)*, one MYB (*Arabidopsis thaliana* At3g06490), one bZIP (*Vitis vinifera),* NAC1 TF (*Solanum lycopersicum),* one HSF (*Vitis vinifera),* WRKY30 protein (*Vitis aestivalis)* and SHORT VEGETATIVE PHASE MADS-box protein (*Arabidopsis thaliana* At2g22540, SVP) (Additional file 17). Abundant genes encoding putative TFs in the AZ support the contention that a key role is played by transcription regulation during abscission in olive [9]. Thus, among all TF genes expressed differentially between the two tissues; only 25 genes were found to be expressed preferentially in ripe fruit and 94 genes in AZ (Additional file 17).

A total of 24 ZF proteins within our analysis show this class of TF to be among the most represented both in ripe fruit and in AZ tissues (Figure 8). Indeed, a ZP gene, *AtZFP2*[37], reportedly has delayed flower senescence as well as abscission, but AtZFP2 has been shown to participate with DNA BINDING WITH ONE FINGER (AtDOF4.7) in suppressing *PGAZAT* expression [20]. According to our data, 16 of out 24 ZF genes (Additional file 17) are among the over-regulated TFs in the olive AZ, supporting the coordinated action of ZF proteins in the AZ during fruit abscission. The majority of bHLH proteins identified to date have been functionally characterized in arabidopsis, but, in other plant species, a low number of *bHLH* genes have been functionally characterized [38]. These genes serve to regulate carpel, anther, and epidermal-cell development, as well as flavonoid biosynthesis, phytochrome signaling, hormone signaling, stress responses, and fruit dehiscence [38]. Gene transcription is known to be regulated by MYB transcription factors in combination with bHLH proteins, which include certain MYC transcription factors. In this sense, MYB and MYC (bHLH) proteins interact to form multi-protein complexes [39]. Reportedly, MYB and bHLH proteins in arabidopsis, cooperate in TTG1-dependent transcriptional regulation [40]. Also, our results demonstrate over-regulation in the olive AZ of 4 out of 5 *MYB* genes identified (Additional file 17), and 15 out of 20 *bHLH* genes identified (Additional file 17). We cannot rule out the possibility that these bHLH proteins, including MYC2 (*Vitis vinifera*), constitute an interaction partner for these MYB TFs for the regulation of genes needed for processes downstream in the AZ during fruit abscission. Further research is necessary to ascertain whether these bHLH TFs act together with MYB proteins in the olive AZ. In this context, homo-and heterodimers formed by bZIP transcription factors are key in the regulation of development and defense responses [41]. Also, bZIP TFs are members of TFs families abundantly represented in the olive AZ (Figure 8). Among those are *HY5* and *RF2a* genes, which were induced in the olive AZ compared with ripe fruit (Additional file 17), and were induced also in melon AZ during early induction of mature-fruit abscission [42]. HY5 is known to mediate the light response [43], whereas RF2a and RF2b functions may be involved in biotic or abiotic stress response or signaling [44]. Three *TGA-type bZIP* genes have been proposed as governing abscission and regulating abscission-related gene expression [45] as well as up-regulation of the genes *bZIP16*, *bZIP17*, *bZIP44*, *bZIP45*, *bZIP53*, and *VIP1* in the olive AZ during mature-fruit abscission [9]. In this light, bZIP proteins appear to be positive regulators in abscission signaling. In addition, most NAC proteins were also overexpressed in the olive AZ in comparison with ripe fruit (Additional file 17). Previously, we have found that 5 genes homologous to NAC TFs (*ANAC029*, *ANAC002*, *ANAC022*, *ANAC091*, and *ANAC042*) showed enhanced expression during mature-fruit abscission [9], as also reported during the immature-fruit abscission in apple [46]. This finding is noteworthy because transcriptome analyses have recently demonstrated regulation by a NAC transcription factor family. This is not restricted to biotic and abiotic stress responses, but also affects numerous other processes, including senescence, ABA signaling and fruit ripening [28, 47].

**Figure 10:**
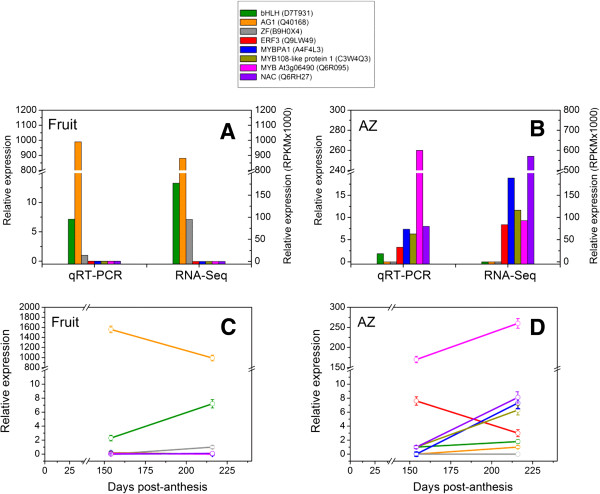
**Validation of pyro-sequencing data.** A total of eight TF genes showing differential expression in our pyro-sequencing experiment were selected and their relative expression determined using qRT-PCR in two olive tissues: **(A)** fruit pericarp at 217 DPA and **(B)** fruit-AZ at 217 DPA. **(C)** qRT-PCR analysis of eight TF genes in olive fruit during fruit ripening. **(D)** qRT-PCR analysis of eight TF genes in olive AZ during abscission of mature-fruit. In the Materials and Methods section, genes and their primers are indicated. Relative expression values were normalized to the lowest expression value taken as 1. The data represent the mean values (±SEs) of duplicate experiments from three independent biological samples.

To validate our RNA-seq results, we performed quantitative real time PCR (qRTPCR) to determine the levels of expression in eight olive genes taken from the list of TF genes differentially expressed across ripe fruit and AZ. Three genes, *bHLH* (UniProt ID: D7T931), *AG1* (UniProt ID: Q40168) and *ZF* (UniProt ID: B9H0X4), were identified as being overexpressed in ripe fruit in RNA-seq data analysis and thus were designated for further confirmation (Figure 10A). Similarly, 5 genes, *ERF3 (*UniProt ID: Q9LW49), *MYBPA1* (UniProt ID: A4F4L3), *MYB108* (UniProt ID: C3W4Q3), *NAC* (UniProt ID: Q6RH27) and MYB/At3g06490 (UniProt ID: Q6R095), were identified as being overexpressed in AZ in RNA-seq data analysis and were assigned to further confirmation (Figure 10B).

The qRT-PCR analysis confirmed the enrichment *bHLH*, *AG1* and *ZF* genes in ripe fruit and the enrichment of *ERF3*, *MYBPA1*, *MYB108*, *NAC* and *MYB/At3g06490* genes in the olive AZ. Notably, the expression of *ERF3*, *MYBPA1*, *MYB108*, *NAC* and *MYB/At3g06490* were not detected in fruit (Figure 10A), and the expression of *bHLH*, *AG1* and *ZF* were not detected in AZ (Figure 10B). Thus, the qRT-PCR expression results correlated with the RNA-seq expression data for the genes tested. In addition, we used qRT-PCR analysis for the expression profiles of eight TFs in olive fruit and AZ during fruit ripening and abscission (between 154 and 217 DPA). The expression of *bHLH* and *ZF* increased 3-fold and 1-fold in olive fruit, respectively, during ripening, while *AG1* expression decreased 1.6-fold during ripening (Figure 10C), implying that these genes are involved in ripening events. On the other hand, transcripts of *MYBPA1*, *MYB108*, *NAC* and *MYB/At3g06490* accumulated during abscission in olive AZ, whereas the expression of *ERF3* was decreased in olive AZ during abscission (Figure 10D). Hence, the expression pattern of some genes in olive fruit or AZ*,* performed by qRT-PCR, are shown to represent the transcriptome related to fruit ripening or the transcriptome related to the activation of abscission.

## Conclusion

We performed 454 transcriptome sequencing and *de novo* assembly for two tissues, ripe fruit and AZ, of *Olea europaea*. As a result, we describe transcriptomic differences between the ripe fruit and this AZ occurring at last stage of ripening in olive as well as potential new genes generated. Changes in gene transcripts were accompanied by changes in expression of TFs, especially those in the TFs MADS-box, ZF, homeobox domain proteins, bHLH, and bZIP families, that putatively may trigger the cross-talk between fruit and AZ. Our results indicate that genes encoding members of Aux/IAA, C2H2L, and CAMTA families were preferentially transcribed in ripe fruit. By contrast, TF genes of the HSF, GRAS, GAGA-binding protein, EIN3/EIL, E2F/DP, CCAAT-binding protein, and WRKY families were preferentially transcribed in AZ. Furthermore, by quantitative real-time PCR analysis, we confirmed the mRNA-Seq results for eight TF genes. This result implies that the study of those TFs associated with the expression pattern observed in ripe fruit could open major biological pathways governing gene-expression regulation in ripe fruit. These data supply the first comprehensive and comparative molecular information for understanding the expression differences in these tissues.

## Methods

### Plant material and RNA isolation

20-year-old olive trees (*Olea europaea* L. cv. Picual) in an orchard near Badajoz (Spain) grown under drip irrigation and fertirrigation (irrigation with suitable fertilizers in the solution) were studied. Picual olive flowers were tagged on the day of pollination and the fruit-pericarp (fruit mesocarp and epicarp) and fruit-AZ samples were collected from olive fruits subsequently harvested at last stage of ripening (217 days post-anthesis, DPA), at which time they abscise (Figure 1). The fruit AZs, located between the pedicel and fruit, were manually dissected from longitudinal sections of the samples with a razor blade into pieces to a maximum width of 1 mm on each side of the abscission fracture plane [15]. Fruit-AZ wings containing pericarp or pedicel/calyx-like tissues were discarded. Fresh samples (fruit-pericarp and fruit-AZ at 217 DPA), using 300 fruits, were immediately frozen in liquid nitrogen and stored at-80°C for RNA isolation.

Total RNA was extracted from fruit-pericarp and-AZ tissues at 217 DPA using the Spectrum Plant Total RNA Kit (Sigma-Aldrich) according to the manufacturer’s instructions and eluted with nuclease-free water. After DNaseI (Ambion) treatment, RNA quality was gel verified and quantified spectrophotometrically (NanoDrop, ThermoScientific, http://http://www.thermofisher.com/). Messenger RNA was isolated twice with Dynabeads Oligo (dT)25 (Dynal Biotech ASA, Dynal Invitrogen, http://www.invitrogen.com) to minimize rRNA contamination. One microgram of mRNA per sample was used as template for first-strand cDNA synthesis using SMART technology (Clontech Laboratories Inc, http://www.clontech.com/) to favor full-length synthesis. Double-stranded cDNA was made by 13 cycles of longdistance PCR. Complementary DNA was purified with QIAquick columns (Qiagen, http://www.qiagen.com/) to eliminate oligo-dT and enzymes. The cDNA quality was verified with an Agilent 2100 Bioanalyzer (Nimblegen, http://www.nimblegen.com/).

### Library preparation for pyro-sequencing

Three micrograms of each cDNA sample were nebulized to produce fragments of a mean size between 400 and 800 bp. Preparation of cDNA fragment libraries and emulsion PCR conditions were performed as described in the Roche GS FLX manual. Pyro-sequencing was performed on a Roche Genome Sequencer FLX instrument (454LifeScience-Roche Diagnostics, http://www.454.com/) at Lifesequencing S.L. (Valencia, Spain).

### Trimming and assembly of pyro-sequenced reads

The quality of the reads was assessed with PERL scripts developed at Lifesequencing for trimming and validation of high-quality sequences. Adaptor sequences used for library preparation were entered in an adaptor-trimming database to the PERL Program. New SFF output files were generated with the sfftools (454 Life Science/Roche), keeping the largest starting trimpoint and the smallest ending trimpoint. Trimmed reads were assembled with NEWBLER version 2.3 (454 Life Science/Roche) with default parameters. Following quality control, when performing the assembly, some reads were removed due to short quality for the reads to be used.

### Annotation

We selected a wide set of reference proteins from taxonomically related organisms. We included all proteins form *eudicotyledons* with annotations for the terms: carbohydrate metabolic process, secondary metabolic process, cell-wall, cell-wall organization, and phytohormones, in order to have a complete reference protein representation for these specific aspects probably related with ripening and abscission process. The total number of reference proteins was 125,428. The inclusion of proteins from taxonomically distant organisms with rich functional annotations such as *Vitis vinifera* or *Ricinus communis,* allowed us to annotate new proteins that could be lost if we include proteins only from close organisms. To obtain a high quality annotation we chose a very restrictive level of similarity between the isotig and the annotator reference protein. The similarity required must be high to sufficiently support the inference of function from the reference protein. In this work, BLAST E value lower than 10^-20^ was required for function inference. It is important to note that the smaller the E value is, the higher similarity between sequences is, and thus, the greater the confidence of the function assignment is. The massive BLASTX of all isotigs against the 125,428 reference proteins was performed using a cloud computing environment (Amazon web services).

### Quantification of the expression levels

The reference proteins were proteins representative of UniRef90 clusters. This strategy fixed a minimum similarity distance between reference proteins and was the basis of our clustering of isotigs for obtaining unigenes and quantifying their expression levels. The name of each unigene was inferred from the name of the UniRef90 representative proteins that annotated each unigene. We quantified the expression for these unigenes, here defined as clusters of isotigs annotated by the same reference protein. The number of reads assigned to each isotig was calculated taking into account that the reads of each contig were counted only one time. Given that isotigs represent transcribed isoforms, it could be possible that different isotigs sharing some contigs were clustered within the same unigene. In those cases, the reads of each contig was counted only one time. The normalization of the absolute values of the number of reads was done based on [48]. We obtained the RPKM (Reads Per Kilobase of exon model per Million mapped reads). In this case, we used the length of the reference protein in nucleotides since we were working without a reference genome and then without exon models. This normalization allows the comparison of the expression values between unigenes from the same or from different samples [48].

### Differential expression analysis

The method used for the analysis of differential expression in this work was edger [49], a Bioconductor package for differential expression analysis of digital gene-expression data able to account for biological variability.

EdgeR models count data using on overdispersed Poisson model, and use an empirical Bayes procedure to moderate the degree of over-dispersion across genes. For the analysis of the differential expression with Edge R the input was a table of counts, with rows corresponding to genes/proteins and columns to samples. EdgeR models the data as negative binomial (NB) distributed, Y_gi_ ~ NB(M_i_p_gj_, *Ф*_g_) for gene g and sample i. Here M_i_ is the library size (total number of reads), *Ф*_g_ is the dispersion, and p_gj_ is the relative abundance of gene g in experimental group j to which sample i belongs. The NB distribution reduces to Poisson when *Ф*_g_ = 0. This is an especially appropriate method to be used in RNA-Seq projects [50, 51]. In this work, an isotig was considered differentially expressed when it exhibited highly significant difference in read abundance at P < 0.01.

### GO annotations

GO annotations [52] were obtained from Uniprot and inferred from the GO annotations of the proteins representative of each unigene. GO Terms coming from the 3 different GO ontologies (Biological process, Molecular function and Cellular component) were analyzed separately. We found the number of proteins annotated with each term. In the GOSlim analysis, every GO term was translated into a GO Term taken from a set of selected general GO Terms in order to provide a more general and homogeneous perspective of the GO Terms found in a sample. To perform the GOSlim analysis, we selected the GOSlim terms proposed by the European Institute of Bioinformatics (EBI) as GO Terms selected for studies in Plants. The GO-slim studies were developed using Bio4j (http://www.bio4j.com/), a graph database that integrates all Uniprot, GO, taxonomy, RefSeq and Enzyme database elements in nodes connected by edges that represent their relationships. We selected a subset of terms to gain a broad functional overview and, using bio4j at the back-end, we obtained the GO-slim results. At this selected granularity level we obtained the functional profile of GO-slim terms that allowed us to highlight general features.

### Quantitative RT-PCR

Total RNA (2 μg) was reverse-transcribed with random hexamers and Superscript III (Invitrogen), according to the manufacturer’s instructions. Purified cDNA (2 ng) was used as a template for qRTPCR. qRT-PCR assays were performed with gene-specific primers. Primer sequences were 5′- CATGTCAGAGCAAAGAGAGGGCAA-3′ (forward) and 5′-ACTCGCTGCTGATAGTTTCAT-3′ (reverse) for *bHLH* (UniProt ID: D7T931); 5′-ATGGCATTGCAGAGTGATCAATCA-3′ (forward) and 5′-TTGAAGAGGTGGTTGATCTTG-3′ (reverse) for *AG1* (UniProt ID: Q40168); 5′-AATGAGGGAATCTGCCATACT-3′ (forward) and 5′-CTCTCTAGCCACGTGGCCAGA-3′ (reverse) for *ZF* (UniProt ID: B9H0X4); 5′-AATGGCGTTAAGGAGGTCCACTAC-3′ (forward) and 5′-AGGTAAAGGGAAGTTAGTTTTAGC-3′ (reverse) for *ERF3 (*UniProt ID: Q9LW49); 5′-ATGGGAAGGTCTCCTTGTTGTTCA-3′ (forward) and 5′-CTTGATCTCATTGTCGGTTCGACC-3′ (reverse) for *MYBPA1* (UniProt ID: A4F4L3); 5′-TATTTACGCCCAGACGTTCGTCGA-3′ (forward) and 5′-TCTCTCAACCAATCGTGGCATCCA-3′ (reverse) for *MYB108* (UniProt ID: C3W4Q3); 5′-CTTGATGATTGGGTGTTGTGCCGA-3′ (forward) and 5′-TTGATCATTGTACTGCATTTGAGA-3′ (reverse) for *NAC* (UniProt ID: Q6RH27); 5′-G TATTTACGCCCAGACGTTCGTCGA-3′ (forward) and 5′-TCTCTCAACCAATCGTGGCATCCA-3′ (reverse) for MYB transcription factor At3g06490 (UniProt ID: Q6R095). The cDNA was amplified using SYBRGreen-PCR Master kit (Applied Biosystems, Foster City, CA, USA) containing an AmpliTaq Gold polymerase on an iCycler (BioRad Munich, Germany), following the protocol provided by the supplier. Samples were subjected to thermal cycling conditions of DNA polymerase activation at 94°C, 45 s at 55°C, 45 s at 72°C, and 45 s at 80°C; a final elongation step of 7 min at 72°C was performed. The melting curve was designed to increase 0.5°C every 10 s from 62°C. The amplicon was analyzed by electrophoresis and sequenced once for identity confirmation. qRT-PCR efficiency was estimated via a calibration dilution curve and slope calculation. Expression levels were determined as the number of cycles needed for the amplification to reach a threshold fixed in the exponential phase of the PCR (CT). The data were normalized for the quantity of *O. europaea ubiquitin* (*OeUB*) gene [53]. Duplicates from three biological replicates were used in two independent experiments.

## Electronic supplementary material

Additional file 1: **Results for the 454 sequencing runs.** (DOCX 11 KB)

Additional file 2: **Summary of parameters used for the sequencing and assembly in the study of the olive transcriptomes: fruit (blue bars) and AZ (green bars) at 217 DPA.** (A) Read-length distribution. A total of 443,811 good-quality sequence reads were obtained from the 2 samples. (B) Contig-length distribution. A total of 19,062 contigs were assembled from 199,075 redundant reads obtained after clustering and assemblage. The average contig length was around 500 bases. (C) Contig-read total distribution from fruit and AZ 454 sequencing data. (D) Isotig-length distribution. (TIFF 1 MB)

Additional file 3: **List of 7,756 transcripts with Unigene ID in our experiment.** (XLSX 1 MB)

Additional file 4: **List of 4,391 differentially expressed genes in our experiment (P < 0.01, group I).** (XLSX 784 KB)

Additional file 5: **Genes overexpressed in the fruit pericarp in our experiment (P < 0.01, group I: Cluster A).** (XLSX 297 KB)

Additional file 6: **Genes overexpressed in the fruit AZ in our experiment (P < 0.01, group I: Cluster B).** (XLSX 525 KB)

Additional file 7: **Subcluster A1, A2, B1 and B2.** (XLSX 782 KB)

Additional file 8: **Proportion of annotated isotigs in each of the samples, and the proportion of annotated isotigs that present functional annotations of Gene Ontology (GO) or that are found annotated with the enzyme commission (EC) number.** (TIFF 327 KB)

Additional file 9: **Pathways identified through KEGG mapping.** (XLSX 13 KB)

Additional file 10: **Graphic representation of the starch and sucrose metabolism pathway by KEGG.** Boxes colored in red represent the EC number of the enzymes encoded by differentially expressed genes generated by this study (fruit at 217 DPA vs. AZ at 217 DPA) that are homologous to genes involved in the starch and sucrose metabolism pathway. (PNG 40 KB)

Additional file 11: **Graphic representation of the amino sugar and nucleotide sugar metabolism pathway by KEGG.** Boxes colored in red represent the EC number of the enzymes encoded by differentially expressed genes generated by this study (fruit at 217 DPA vs. AZ at 217 DPA) that are homologous to genes involved in the amino sugar and nucleotide sugar metabolism pathway. (PNG 66 KB)

Additional file 12: **Graphic representation of the cysteine and methionine metabolism pathway by KEGG.** Boxes colored in red represent the EC number of the enzymes encoded by differentially expressed genes generated by this study (fruit at 217 DPA vs. AZ at 217 DPA) that are homologous to genes involved in the cysteine and methionine metabolism pathway. (PNG 45 KB)

Additional file 13: **Graphic representation of the methane metabolism pathway by KEGG.** Boxes colored in red represent the EC number of the enzymes encoded by differentially expressed genes generated by this study (fruit at 217 DPA vs. AZ at 217 DPA) that are homologous to genes involved in the methane metabolism pathway. (PNG 72 KB)

Additional file 14: **Graphic representation of the glycolysis/gluconeogenesis pathway by KEGG.** Boxes colored in red represent the EC number of the enzymes encoded by differentially expressed genes generated by this study (fruit at 217 DPA vs. AZ at 217 DPA) that are homologous to genes involved in the glycolysis/gluconeogenesis pathway. (PNG 26 KB)

Additional file 15: **Graphic representation of the glycine, serine and threonine metabolism pathway by KEGG.** Boxes colored in red represent the EC number of the enzymes encoded by differentially expressed genes generated by this study (fruit at 217 DPA vs. AZ at 217 DPA) that are homologous to genes involved in the glycine, serine and threonine metabolism pathway. (PNG 42 KB)

Additional file 16: **Graphic representation of the arginine and proline metabolism pathway by KEGG.** Boxes colored in red represent the EC number of the enzymes encoded by differentially expressed genes generated by this study (fruit at 217 DPA vs. AZ at 217 DPA) that are homologous to genes involved in the arginine and proline metabolism pathway. (PNG 64 KB)

Additional file 17: **Fruit-or AZ-enriched transcription factors at the last stage of olive fruit ripening.** Sequences were selected after establishing a P < 0.01. The table shows the total read count in RPKMx1000 for each gene after normalization across the 2 samples: (a) fruit at 217 DPA, (b) AZ at 217 DPA. (DOCX 43 KB)

## Abbreviations

AZ: Abscission zone
cDNA: Complementary deoxyribonucleic acid
DPA: Days post-anthesis
qRT-PCR: Quantitative real time polymerase chain reaction
FC: Fold change
GO: Gene ontology
EST: Expressed sequence tag
RPKM: Reads per kilobase of exon per million mapped reads.
