# Supplementary material for: Comparative transcriptional profiling analysis of olive ripe-fruit pericarp and abscission zone tissues shows expression differences and distinct patterns of transcriptional regulation
Source: BMC Genomics. 2013 Dec 9;14(1):866. doi: 10.1186/1471-2164-14-866 (PMC4046656; doi:10.1186/1471-2164-14-866)
Supplement: Supplementary file 1 — Additional file 1: Results for the 454 sequencing runs. (DOCX 11 KB) [file 12864_2013_5569_MOESM1_ESM.docx]

**Additional file 1** Results for the 454 sequencing runs

| **Data** | **Fruit at 217 DPA** | **AZ at 217 DPA** |
| --- | --- | --- |
| Raw reads | 216,418 | 207,078 |
| Raw nucleotides | 76,644,620 | 78,507,372 |
| Clean and processed reads | 199,075 | 198,382 |
| Clean nucleotides | 72,866,923 | 75,914,989 |
| Total number of contigs | 7,681 | 11,381 |
| Average contig size | 889 | 921 |
| Total number of isotigs | 7,003 | 10,045 |
| Total number of annotated isotigs | 6,533 | 9,138 |
| UniProt identities | 4,219 | 5,920 |
| UniProt identities annotated with GO | 3,055 | 4,378 |
| UniProt identities annotated with EC number | 615 | 827 |
