# Supplementary figures and images for: Comparative transcriptional profiling analysis of olive ripe-fruit pericarp and abscission zone tissues shows expression differences and distinct patterns of transcriptional regulation
Source: BMC Genomics. 2013 Dec 9;14(1):866. doi: 10.1186/1471-2164-14-866 (PMC4046656; doi:10.1186/1471-2164-14-866)

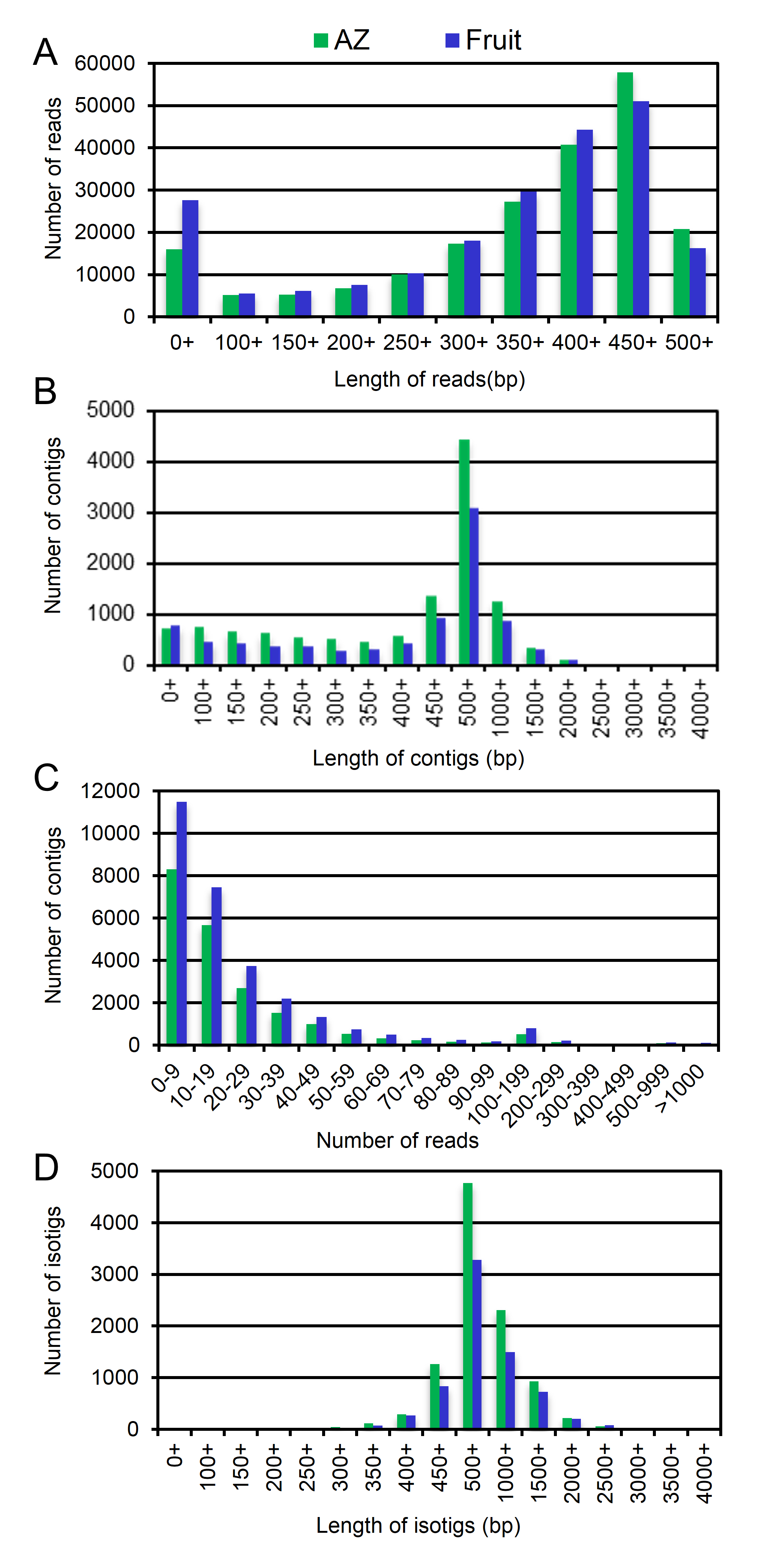

Supplement: Supplementary file 2 — Additional file 2: Summary of parameters used for the sequencing and assembly in the study of the olive transcriptomes: fruit (blue bars) and AZ (green bars) at 217 DPA. (A) Read-length distribution. A total of 443,811 good-quality sequence reads were obtained from the 2 samples. (B) Contig-length distribution. A total of 19,062 contigs were assembled from 199,075 redundant reads obtained after clustering and assemblage. The average contig length was around 500 bases. (C) Contig-read total distribution from fruit and AZ 454 sequencing data. (D) Isotig-length distribution. (TIFF 1 MB) [file 12864_2013_5569_MOESM2_ESM.tiff]

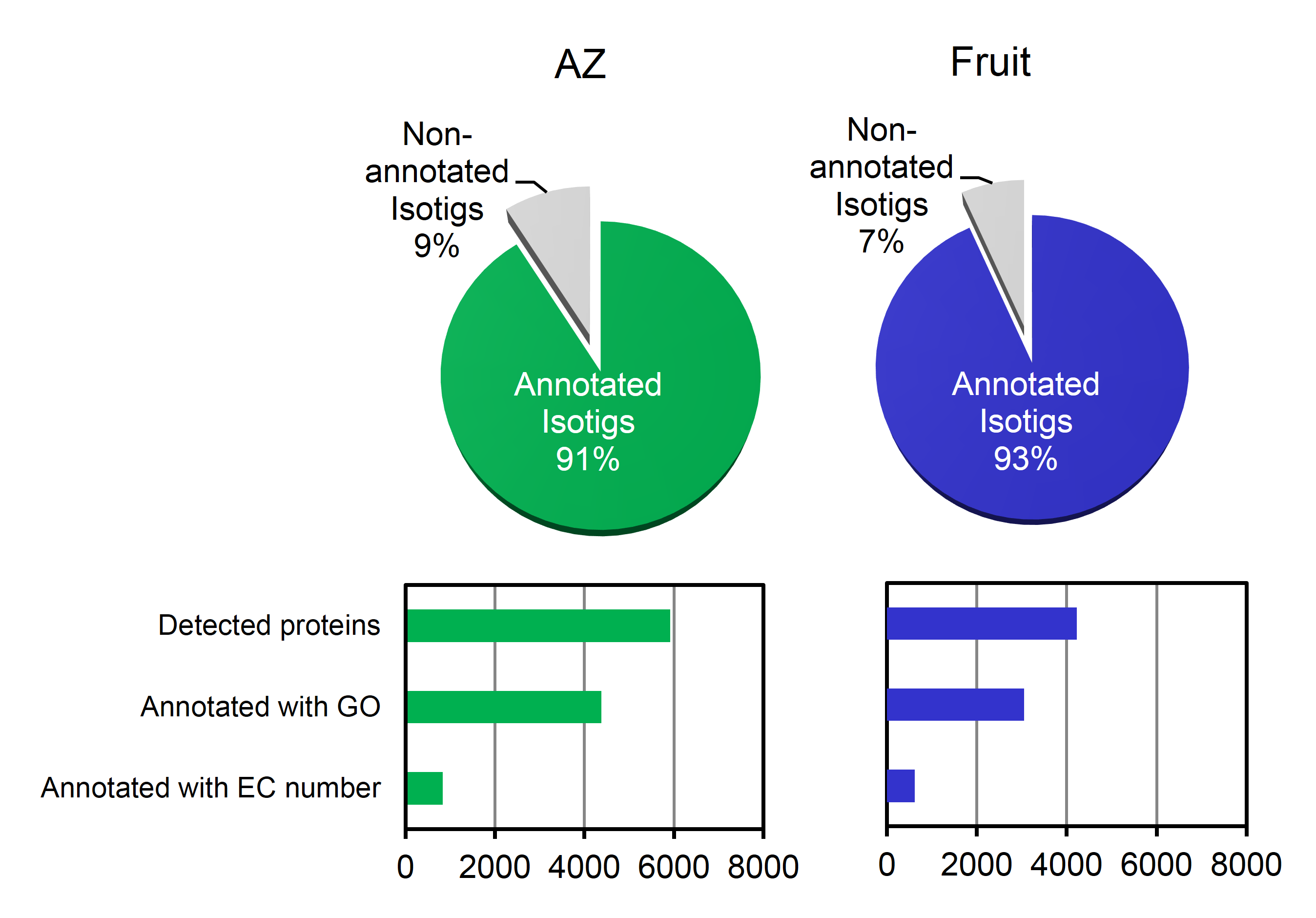

Supplement: Supplementary file 8 — Additional file 8: Proportion of annotated isotigs in each of the samples, and the proportion of annotated isotigs that present functional annotations of Gene Ontology (GO) or that are found annotated with the enzyme commission (EC) number. (TIFF 327 KB) [file 12864_2013_5569_MOESM8_ESM.tiff]

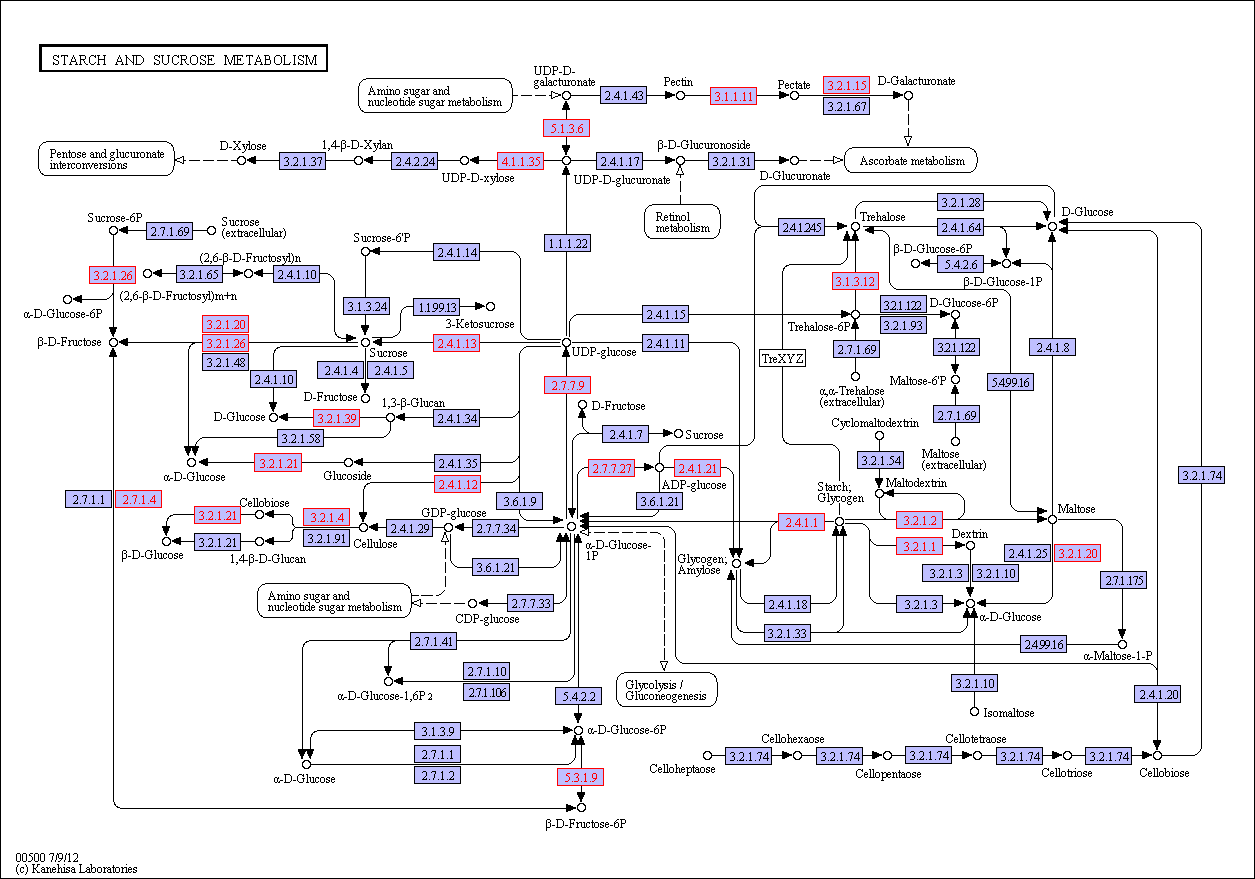

Supplement: Supplementary file 10 — Additional file 10: Graphic representation of the starch and sucrose metabolism pathway by KEGG. Boxes colored in red represent the EC number of the enzymes encoded by differentially expressed genes generated by this study (fruit at 217 DPA vs. AZ at 217 DPA) that are homologous to genes involved in the starch and sucrose metabolism pathway. (PNG 40 KB) [file 12864_2013_5569_MOESM10_ESM.png]

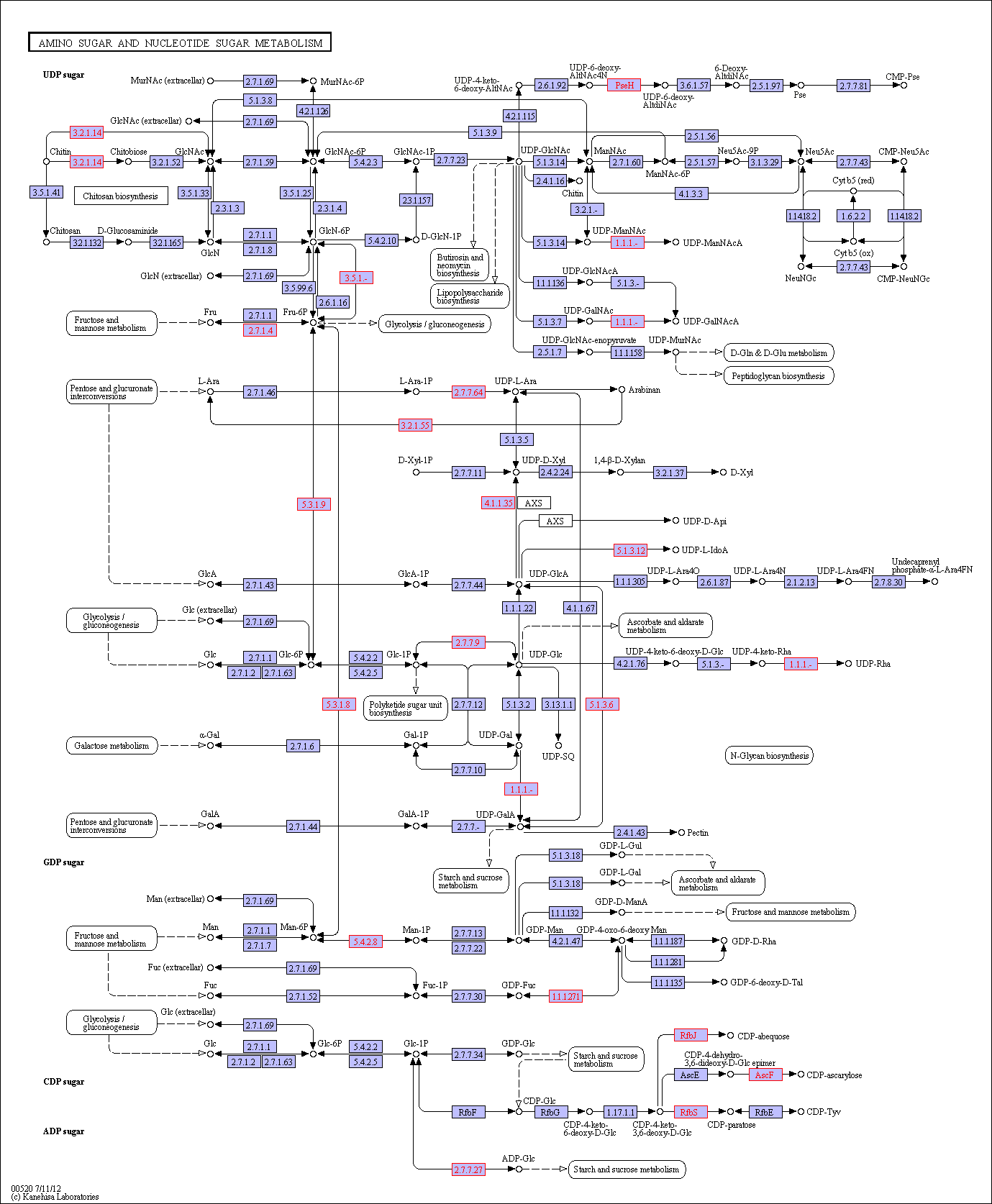

Supplement: Supplementary file 11 — Additional file 11: Graphic representation of the amino sugar and nucleotide sugar metabolism pathway by KEGG. Boxes colored in red represent the EC number of the enzymes encoded by differentially expressed genes generated by this study (fruit at 217 DPA vs. AZ at 217 DPA) that are homologous to genes involved in the amino sugar and nucleotide sugar metabolism pathway. (PNG 66 KB) [file 12864_2013_5569_MOESM11_ESM.png]

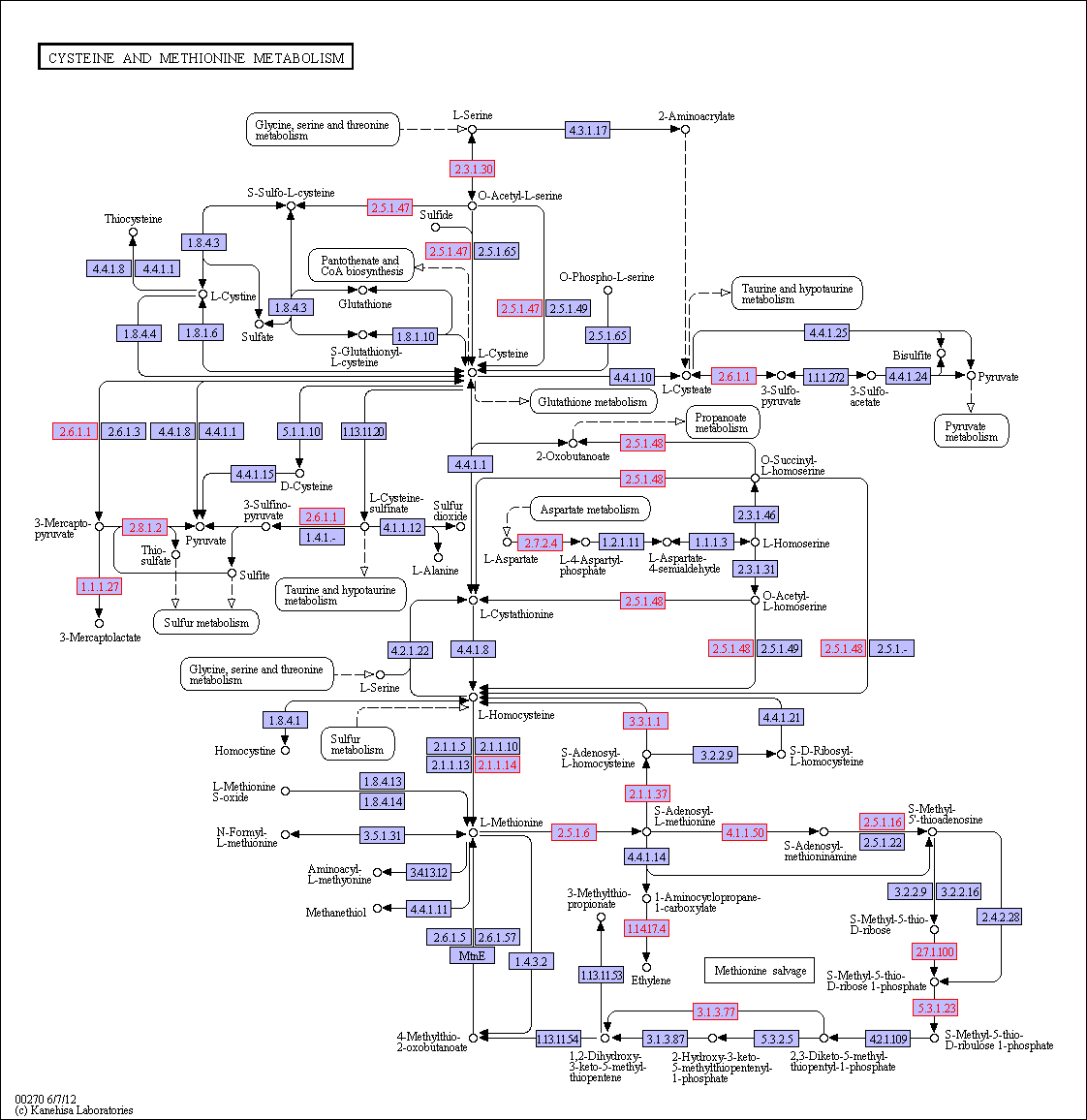

Supplement: Supplementary file 12 — Additional file 12: Graphic representation of the cysteine and methionine metabolism pathway by KEGG. Boxes colored in red represent the EC number of the enzymes encoded by differentially expressed genes generated by this study (fruit at 217 DPA vs. AZ at 217 DPA) that are homologous to genes involved in the cysteine and methionine metabolism pathway. (PNG 45 KB) [file 12864_2013_5569_MOESM12_ESM.png]

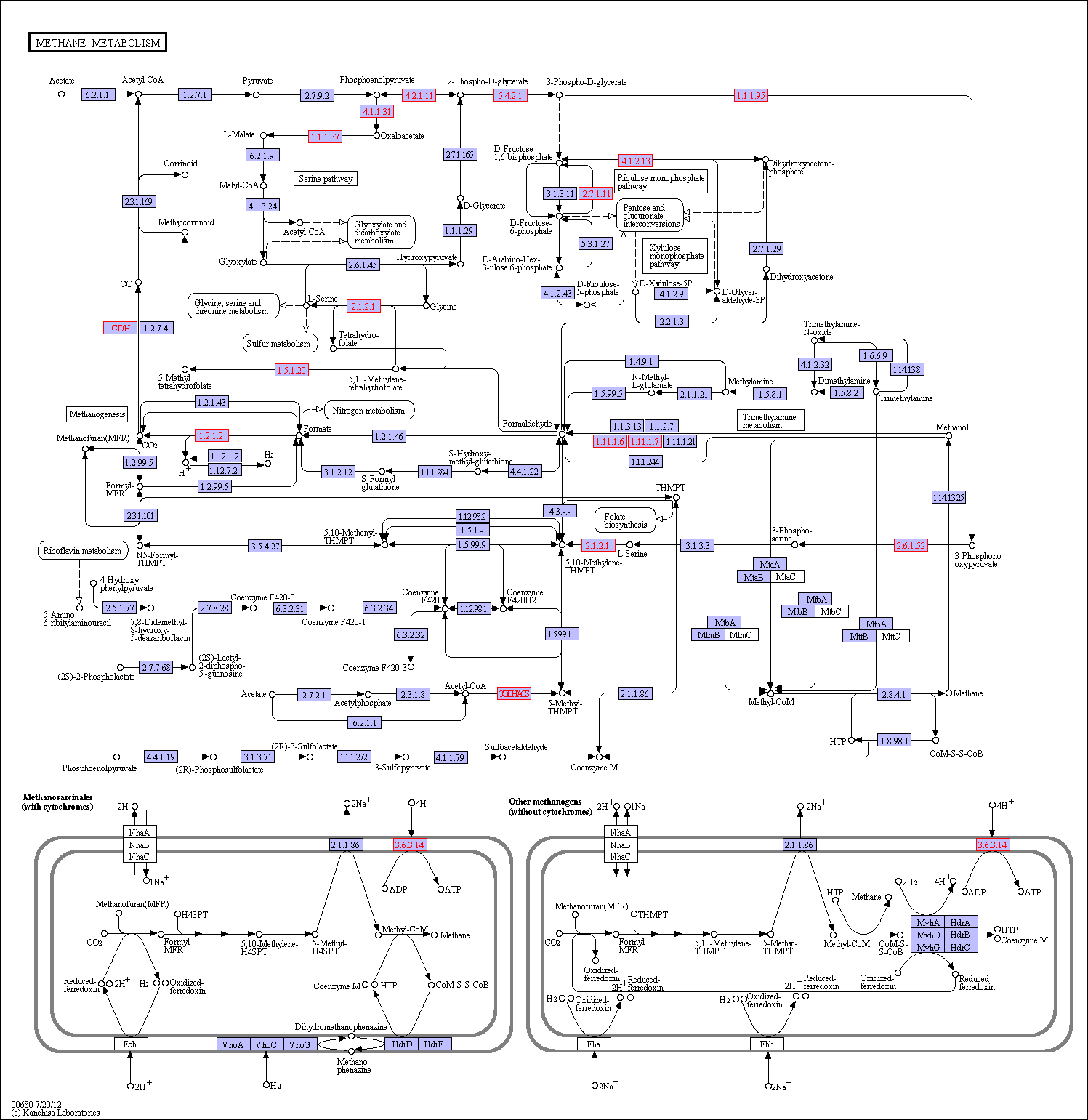

Supplement: Supplementary file 13 — Additional file 13: Graphic representation of the methane metabolism pathway by KEGG. Boxes colored in red represent the EC number of the enzymes encoded by differentially expressed genes generated by this study (fruit at 217 DPA vs. AZ at 217 DPA) that are homologous to genes involved in the methane metabolism pathway. (PNG 72 KB) [file 12864_2013_5569_MOESM13_ESM.png]

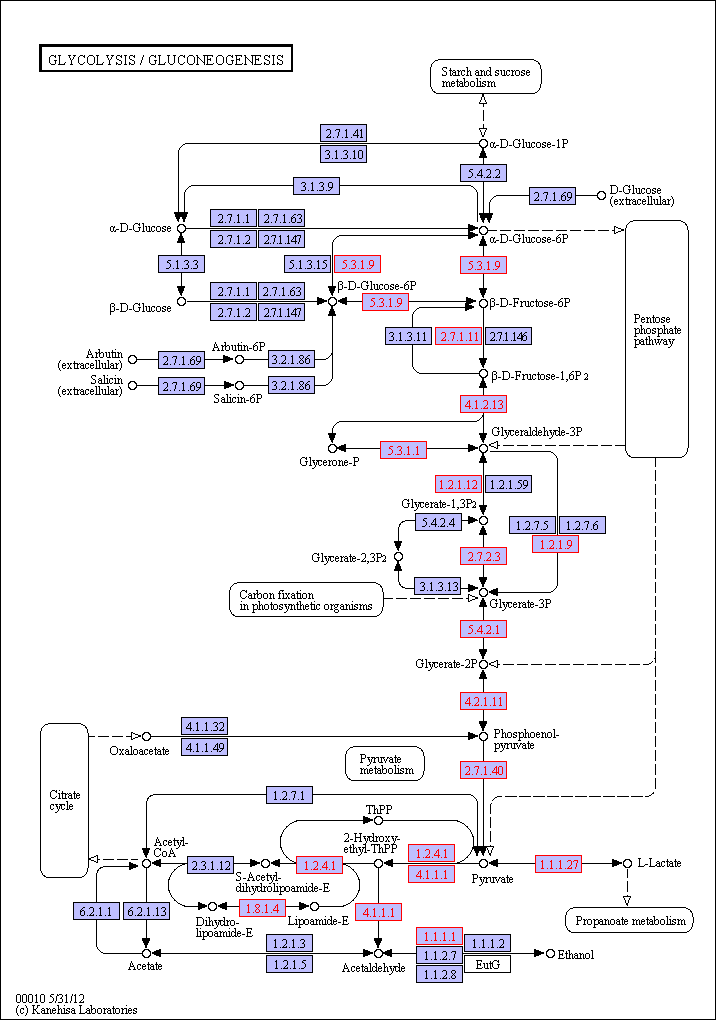

Supplement: Supplementary file 14 — Additional file 14: Graphic representation of the glycolysis/gluconeogenesis pathway by KEGG. Boxes colored in red represent the EC number of the enzymes encoded by differentially expressed genes generated by this study (fruit at 217 DPA vs. AZ at 217 DPA) that are homologous to genes involved in the glycolysis/gluconeogenesis pathway. (PNG 26 KB) [file 12864_2013_5569_MOESM14_ESM.png]

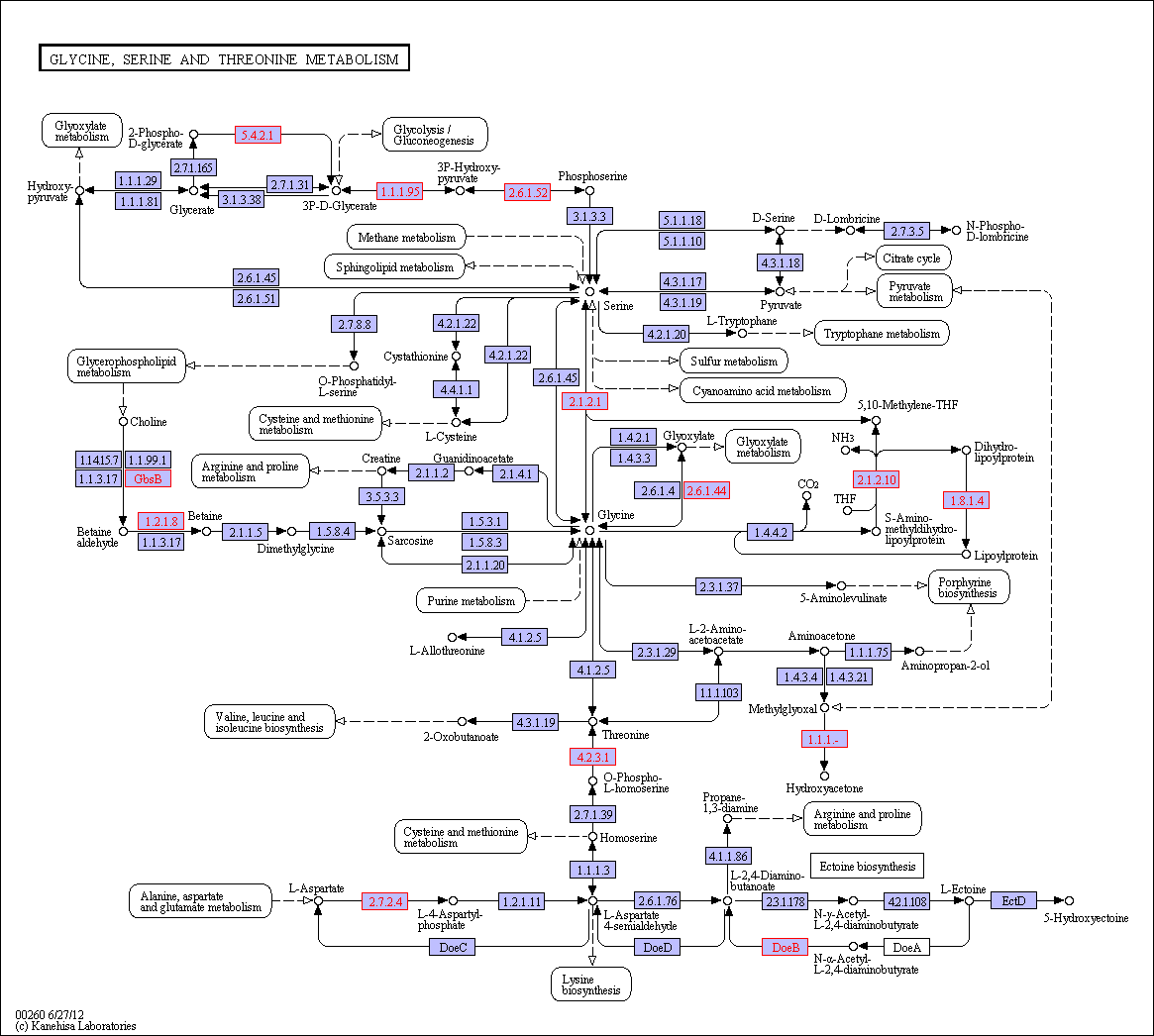

Supplement: Supplementary file 15 — Additional file 15: Graphic representation of the glycine, serine and threonine metabolism pathway by KEGG. Boxes colored in red represent the EC number of the enzymes encoded by differentially expressed genes generated by this study (fruit at 217 DPA vs. AZ at 217 DPA) that are homologous to genes involved in the glycine, serine and threonine metabolism pathway. (PNG 42 KB) [file 12864_2013_5569_MOESM15_ESM.png]

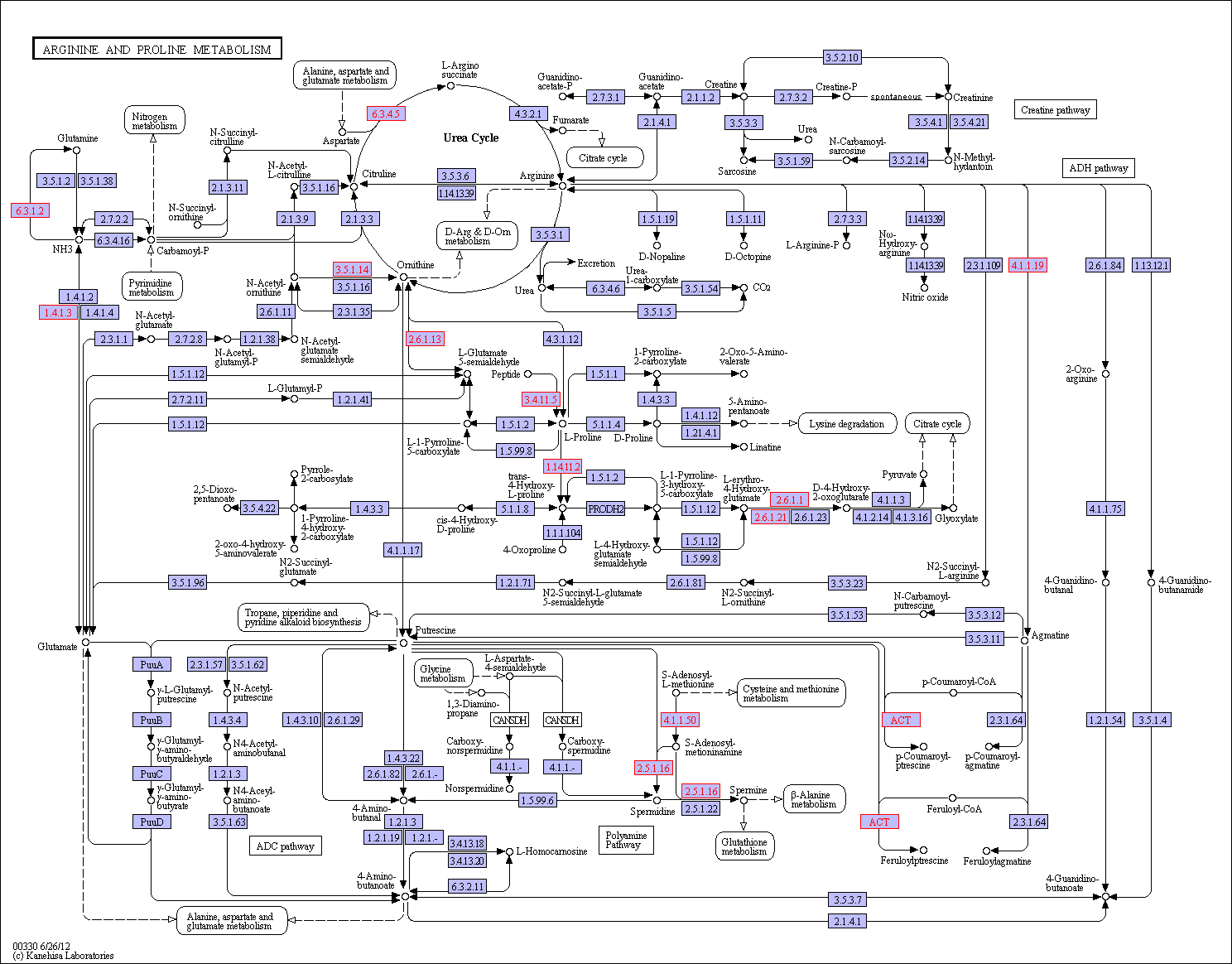

Supplement: Supplementary file 16 — Additional file 16: Graphic representation of the arginine and proline metabolism pathway by KEGG. Boxes colored in red represent the EC number of the enzymes encoded by differentially expressed genes generated by this study (fruit at 217 DPA vs. AZ at 217 DPA) that are homologous to genes involved in the arginine and proline metabolism pathway. (PNG 64 KB) [file 12864_2013_5569_MOESM16_ESM.png]
