# Supplementary material for: Comparative transcriptional profiling analysis of olive ripe-fruit pericarp and abscission zone tissues shows expression differences and distinct patterns of transcriptional regulation
Source: BMC Genomics. 2013 Dec 9;14(1):866. doi: 10.1186/1471-2164-14-866 (PMC4046656; doi:10.1186/1471-2164-14-866)
Supplement: Supplementary file 17 — Additional file 17: Fruit-or AZ-enriched transcription factors at the last stage of olive fruit ripening. Sequences were selected after establishing a P < 0.01. The table shows the total read count in RPKMx1000 for each gene after normalization across the 2 samples: (a) fruit at 217 DPA, (b) AZ at 217 DPA. (DOCX 43 KB) [file 12864_2013_5569_MOESM17_ESM.docx]

**Additional file 17:** Fruit- or AZ-enriched transcription factors at the last stage of olive fruit ripening and abscission. Sequences were selected after establishing a P<0.01. The table shows the total read count in RPKMx1000 for each gene after normalization across the 2 samples: (a) fruit-pericarp at 217 DPA, (b) AZ at 217 DPA.

|  | **UniProt ID** | **Fruit** | **AZ** | **p.value** | **Description** | |
| --- | --- | --- | --- | --- | --- | --- |
| Cluster A |  |  |  |  |  | |
| Cluster A1 | Enriched in fruit |  |  |  |  | |
|  | Q8H278 | 330.62 | 36.59 | 7.42E-41 | MADS-box protein, TAGL2 = *Solanum lycopersicum* | |
|  | Q03489 | 269.71 | 24.90 | 1.10E-35 | MADS-box protein, AGL9, Floral-binding protein 2, FBP2 = *Petunia hybrida* | |
|  | B9S9F0 | 118.44 | 16.92 | 2.93E-21 | Transcription factor = *Ricinus communis* | |
|  | E0CPR7 | 78.36 | 5.22 | 1.00E-15 | Transcription factor = *Vitis vinifera* | |
|  | B9SDI4 | 64.31 | 15.22 | 3.31E-14 | Homeobox protein, Bel1 = *Ricinus communis* | |
|  | A5B7D8 | 43.66 | 4.92 | 1.38E-12 | Zinc finger protein = *Vitis vinifera* | |
|  | B9T724 | 74.28 | 11.14 | 1.85E-12 | GATA transcription factor = *Ricinus communis* | |
|  | B9SZ77 | 85.36 | 23.55 | 4.16E-11 | bZIP transcription factor, Light-inducible protein CPRF-2 = *Ricinus communis* | |
|  | B9SCP7 | 149.20 | 46.03 | 4.99E-08 | Zinc-finger protein, Dc50 = *Ricinus communis* | |
|  | D7UCZ6 | 199.67 | 77.56 | 2.28E-07 | NAC protein = *Vitis vinifera* | |
|  | B9SMN9 | 86.78 | 13.80 | 6.87E-07 | Transcription factor = *Ricinus communis* | |
|  | Q9SXS8 | 168.89 | 100.74 | 4.60E-03 | Ethylene-responsive transcription factor 3, NtERF3 = *Nicotiana tabacum* | |
| Cluster A2 | Fruit-specific transcription factors |  |  |  |  | |
|  | Q40168 | 880.38 | 0.00 | 1.21E-182 | MADS-box protein, AG1 = *Solanum lycopersicum* | |
|  | O22300 | 103.29 | 0.00 | 3.82E-37 | Homeobox protein knotted-1-like, LET12 = *Solanum lycopersicum* | |
|  | D7T931 | 177.04 | 0.00 | 5.92E-36 | basic helix-loop-helix (bHLH) transcription factor = *Vitis vinifera* | |
|  | Q7Y0Z7 | 46.26 | 0.00 | 4.85E-27 | Homeobox protein, Bell-like homeodomain protein 2, BL2 = *Solanum lycopersicum* | |
|  | D7SY02 | 64.76 | 0.00 | 1.90E-26 | Homeobox protein = *Vitis vinifera* | |
|  | B9H0X4 | 95.04 | 0.00 | 6.78E-21 | Zinc finger protein = *Populus trichocarpa* | |
|  | B9SGM7 | 70.77 | 0.00 | 6.78E-21 | bZIP transcription factor, Transcription factor RF2a = *Ricinus communis* | |
|  | E0CPI0 | 74.75 | 0.00 | 1.73E-18 | Zinc finger protein = *Vitis vinifera* | |
|  | Q84N38 | 31.57 | 0.00 | 8.88E-16 | PHD-type zinc finger = *Nicotiana benthamiana* | |
|  | Q0EDB1 | 49.42 | 0.00 | 1.78E-15 | MYB transcription factor, VvMYBA22-cs = *Vitis vinifera* | |
|  | Q9SJM6 | 99.37 | 0.00 | 1.42E-14 | Zinc finger A20 and AN1 domain-containing stress-associated protein 4 (AtSAP4), SAP4 At2g36320 | |
|  | D7SH59 | 40.11 | 0.00 | 9.09E-13 | bHLH transcription factor = *Vitis vinifera* | |
|  | A5C5T1 | 35.04 | 0.00 | 5.82E-11 | Homeobox protein = *Vitis vinifera* | |
|  | B9RUW0 | 50.69 | 0.00 | 2.33E-10 | Aux/IAA, IAA1 = *Ricinus communis* | |
|  | E0CTE8 | 32.56 | 0.00 | 9.54E-07 | bHLH transcription factor = *Vitis vinifera* | |
|  | D9ZIP8 | 8.58 | 0.00 | 4.77E-07 | bZIP transcription factor, BZIP1 = *Malus domestica* | |
|  | D9ZIV7 | 13.02 | 0.00 | 7.63E-06 | CAMTA domain class transcription factor, CAMTA1 = *Malus domestica* | |
|  | B9S5M4 | 16.08 | 0.00 | 9.77E-04 | NAC transcription factor = *Ricinus communis* | |
|  | D7U0I3 | 5.33 | 0.00 | 4.88E-04 | Homeobox protein = *Vitis vinifera* | |
|  | Q9LYB9 | 23.29 | 0.00 | 2.44E-04 | CW-type zinc finger, Methyl-CpG-binding protein MBD4, MBD4 At3g63030 | |
|  | E0CT50 | 11.25 | 0.00 | 7.81E-03 | bHLH transcription factor = *Vitis vinifera* | |
|  | D9ZIS2 | 4.95 | 0.00 | 7.81E-03 | C2H2L domain class transcription factor, C2H2L12 = *Malus domestica* | |
|  | D7UCK3 | 6.98 | 0.00 | 3.91E-03 | bZIP transcription factor = *Vitis vinifera* | |
|  | A5B890 | 6.73 | 0.00 | 3.91E-03 | bHLH transcription factor = *Vitis vinifera* | |
|  | D7TEZ0 | 7.19 | 0.00 | 1.95E-03 | Zinc finger protein = *Vitis vinifera* | |
| Cluster B1 | Enriched in AZ |  |  |  |  | |
|  | B9SJN1 | 4.75 | 235.99 | 1.57E-143 | bZIP transcription factor HY5 = *Ricinus communis* | |
|  | Q84QD4 | 18.34 | 166.13 | 3.87E-62 | EIN3/EIL = *Nicotiana tabacum* | |
|  | A5C9F3 | 105.31 | 348.79 | 1.61E-37 | Homeobox protein = *Vitis vinifera* | |
|  | B2Z454 | 6.83 | 157.00 | 3.21E-36 | GAGA-binding transcriptional activator, BBR/BPC1-like = *Vitis vinifera* | |
|  | Q6L467 | 9.77 | 99.89 | 5.11E-20 | Homeobox protein, HAT7 = *Solanum demissum* | |
|  | B9RFW3 | 45.93 | 173.23 | 2.92E-16 | NAC protein = *Ricinus communis* | |
|  | A5BSP9 | 49.02 | 225.49 | 4.72E-14 | bHLH transcription factor = *Vitis vinifera* | |
|  | B9SQ16 | 46.03 | 138.08 | 3.18E-10 | Phd/F-box containing protein = *Ricinus communis* | |
|  | D7TNF1 | 5.19 | 40.50 | 9.33E-09 | Homeobox protein = *Vitis vinifera* | |
|  | D7TRM3 | 109.52 | 215.48 | 1.07E-09 | Homeobox protein = *Vitis vinifera* | |
|  | C1KH72 | 5.90 | 57.52 | 1.71E-08 | AP2/ERF protein, RAP2-like protein = *Juglans nigra* | |
|  | B9GKQ8 | 22.75 | 72.81 | 3.04E-07 | Homeobox protein = *Populus trichocarpa* | |
|  | B9IPK2 | 11.17 | 55.86 | 4.13E-05 | Zinc finger protein = *Populus trichocarpa* | |
|  | B9R8U7 | 70.81 | 117.65 | 1.85E-04 | bZIP transcription factor = *Ricinus communis* | |
|  | B9RFB9 | 9.30 | 26.35 | 3.46E-04 | Zinc finger protein = *Ricinus communis* | |
|  | Q9LHJ8 | 66.66 | 127.08 | 5.88E-04 | Zinc finger A20 and AN1 domain-containing stress-associated protein 5 (AtSAP5), SAP5 At3g12630 | |
|  | B9SA09 | 65.25 | 99.29 | 9.34E-03 | bHLH transcription factor = *Ricinus communis* | |
|  | B9R762 | 13.56 | 32.77 | 2.89E-03 | R2r3-myb transcription factor = *Ricinus communis* | |
|  | Q00LP0 | 6.87 | 15.32 | 5.40E-03 | GRAS10 = *Solanum lycopersicum* |  |
| Cluster B2 | AZ-specific transcription factors |  |  |  |  | |
|  | A4F4L3 | 0.00 | 188.81 | 5.35E-51 | MYBPA1 protein = *Vitis vinifera* | |
|  | B9SRT4 | 0.00 | 163.64 | 5.35E-51 | WRKY transcription factor = *Ricinus communis* | |
|  | C3W4Q3 | 0.00 | 116.56 | 7.70E-34 | R2R3 transcription factor, MYB108-like protein 1 = *Vitis vinifera* | |
|  | B9SZ17 | 0.00 | 67.05 | 6.16E-33 | Zinc finger protein = *Ricinus communis* | |
|  | Q6R095 | 0.00 | 92.88 | 2.02E-28 | MYB transcription factor, At3g06490 | |
|  | D7TTQ2 | 0.00 | 71.03 | 8.08E-28 | bZIP transcription factor = *Vitis vinifera* | |
|  | Q6RH27 | 0.00 | 570.32 | Q6RH27 | NAC protein, SlNAC1 = *Solanum lycopersicum* | |
|  | D7SVY0 | 0.00 | 88.58 | 3.31E-24 | Heat shock factor protein, HSF = *Vitis vinifera* | |
|  | Q6R7N3 | 0.00 | 68.77 | 5.29E-23 | WRKY transcription factor 30, WRKY30 = *Vitis aestivalis* | |
|  | Q9FVC1 | 0.00 | 98.61 | 1.06E-22 | MADS-box protein, SHORT VEGETATIVE PHASE, SVP, At2g22540 | |
|  | O64647 | 0.00 | 63.67 | 8.47E-22 | TCP-domain, TCP9, At2g45680 | |
|  | Q9LW49 | 0.00 | 83.70 | 3.47E-18 | AP2/ERF, NsERF3 = *Nicotiana sylvestris* | |
|  | A5C907 | 0.00 | 37.72 | 1.39E-17 | bHLH transcription factor = *Vitis vinifera* | |
|  | D1MDP9 | 0.00 | 72.87 | 2.78E-17 | MADS-box protein, Fruitful, FUL = *Vitis vinifera* | |
|  | A5AUF1 | 0.00 | 57.67 | 4.44E-16 | bHLH transcription factor = *Vitis vinifera* | |
|  | D9ZIP2 | 0.00 | 21.23 | 4.44E-16 | bHLH transcription factor, BHLH2 = *Malus domestica* | |
|  | B9RNB2 | 0.00 | 52.58 | 4.44E-16 | WRKY transcription factor = *Ricinus communis* | |
|  | A5C631 | 0.00 | 55.55 | 4.44E-16 | Zinc finger protein = *Vitis vinifera* | |
|  | E0CNW5 | 0.00 | 45.94 | 3.55E-15 | Homeobox protein = *Vitis vinifera* | |
|  | B9SSS9 | 0.00 | 27.28 | 7.11E-15 | WRKY transcription factor = *Ricinus communis* | |
|  | D7TDJ7 | 0.00 | 44.03 | 1.14E-13 | Homeobox protein = *Vitis vinifera* | |
|  | D7T311 | 0.00 | 34.57 | 1.14E-13 | CCAAT-binding factor = *Vitis vinifera* | |
|  | B9RNE6 | 0.00 | 87.96 | 1.82E-12 | bZIP transcription factor = *Ricinus communis* | |
|  | Q84N37 | 0.00 | 24.69 | 1.82E-12 | PHD-type zinc finger = *Pisum sativum* | |
|  | D9ZJ83 | 0.00 | 32.38 | 2.91E-11 | MYBR4 = *Malus domestica* | |
|  | D7SQJ7 | 0.00 | 39.22 | 2.91E-11 | bHLH transcription factor = *Vitis vinifera* | |
|  | B2G283 | 0.00 | 32.54 | 5.82E-11 | WRKY transcription factor 11, WRKY11 = *Vitis thunbergii* | |
|  | B9R9E1 | 0.00 | 30.96 | 9.31E-10 | Heat shock factor protein, HSF = *Ricinus communis* | |
|  | B9SVE1 | 0.00 | 37.30 | 4.66E-10 | Homeobox protein = *Ricinus communis* | |
|  | D9ZJ91 | 0.00 | 19.10 | 4.66E-10 | NAC protein, NAC13 = *Malus domestica* | |
|  | B9SU10 | 0.00 | 22.75 | 1.86E-09 | bZIP transcription factor, RF2a = *Ricinus communis* | |
|  | D1MDP8 | 0.00 | 38.73 | 3.73E-09 | MADS-box protein, Apetala1, AP1 = *Vitis vinifera* | |
|  | B2G284 | 0.00 | 16.03 | 1.86E-09 | WRKY transcription factor 7, WRKY7 = *Vitis thunbergii* | |
|  | D8VD38 | 0.00 | 35.15 | 1.86E-09 | AP2/ERF, ERF11 = *Actinidia deliciosa* | |
|  | Q00LP3 | 0.00 | 17.17 | 1.86E-09 | GRAS6 = *Solanum lycopersicum* | |
|  | Q9LRC7 | 0.00 | 24.76 | 1.49E-08 | bZIP transcription factor, RSG = *Nicotiana tabacum* | |
|  | A5BP13 | 0.00 | 18.68 | 2.98E-08 | bZIP transcription factor = *Vitis vinifera* | |
|  | A5APP5 | 0.00 | 6.66 | 2.98E-08 | Zinc finger protein = *Vitis vinifera* | |
|  | A5BE46 | 0.00 | 6.14 | 2.98E-08 | Zinc finger protein = *Vitis vinifera* | |
|  | O04136 | 0.00 | 18.74 | 5.96E-08 | Homeobox protein knotted-1-like 3, KNAP3 = *Malus domestica* | |
|  | D7T9Z7 | 0.00 | 27.43 | 9.54E-07 | MADS-box protein = *Vitis vinifera* | |
|  | B6GVA3 | 0.00 | 25.73 | 2.38E-07 | Homeodomain leucine zipper protein, hb-1 = *Solanum lycopersicum* | |
|  | B9GS74 | 0.00 | 23.33 | 4.77E-07 | bHLH transcription factor = *Populus trichocarpa* | |
|  | B9S8Z7 | 0.00 | 24.91 | 4.77E-07 | NAC protein = *Ricinus communis* | |
|  | B9SVD5 | 0.00 | 21.10 | 9.54E-07 | NAC protein 21/22 = *Ricinus communis* | |
|  | Q9SQK8 | 0.00 | 22.49 | 2.38E-07 | bHLH transcription factor, Jasmonic acid 3, LEJA3 = *Solanum lycopersicum* | |
|  | D7T487 | 0.00 | 25.17 | 4.77E-07 | Zinc finger protein = *Vitis vinifera* | |
|  | Q9FNZ2 | 0.00 | 17.05 | 2.38E-07 | Zinc finger CCCH domain-containing protein 48 (AtC3H48) (Zinc finger CCCH domain and WD40, ZFWD1 At4g25440 | |
|  | Q8LLR1 | 0.00 | 25.96 | 1.91E-06 | MADS-box protein 3, MADS3 = *Vitis vinifera* | |
|  | D7SH01 | 0.00 | 25.53 | 3.81E-06 | MADS-box protein = *Vitis vinifera* | |
|  | B2G282 | 0.00 | 10.71 | 7.63E-06 | WRKY transcription factor 5, WRKY5 = *Vitis thunbergii* | |
|  | B9SND3 | 0.00 | 25.13 | 1.91E-06 | Zinc finger protein = *Ricinus communis* | |
|  | D7TM37 | 0.00 | 20.40 | 3.81E-06 | Zinc finger protein = *Vitis vinifera* | |
|  | Q9FUY6 | 0.00 | 20.13 | 1.53E-05 | MADS-box protein, JOINTLESS, LeMADS J = *Solanum lycopersicum* | |
|  | B9T7C9 | 0.00 | 16.43 | 6.10E-05 | bZIP transcription factor = *Ricinus communis* | |
|  | D7TIQ1 | 0.00 | 10.35 | 6.10E-05 | bZIP transcription factor = *Vitis vinifera* | |
|  | B9S4V3 | 0.00 | 9.28 | 6.10E-05 | bHLH transcription factor = *Ricinus communis* | |
|  | B9S4T8 | 0.00 | 14.16 | 3.05E-05 | WRKY transcription factor = *Ricinus communis* | |
|  | B9S232 | 0.00 | 10.80 | 1.53E-05 | Heat shock factor protein, HSF8 = *Ricinus communis* | |
|  | A6ZIC0 | 0.00 | 17.94 | 6.10E-05 | C2H2-type zinc finger protein (Cold zinc finger protein 1) = *Solanum lycopersicum* | |
|  | B9RWY6 | 0.00 | 17.21 | 4.88E-04 | bHLH transcription factor = *Ricinus communis* | |
|  | D7TEQ3 | 0.00 | 14.95 | 9.77E-04 | bHLH transcription factor = *Vitis vinifera* | |
|  | O24160 | 0.00 | 9.50 | 1.22E-04 | bZIP transcription factor, GACG-sequence-specific, TGA21 = *Nicotiana tabacum* | |
|  | D7U1V4 | 0.00 | 11.30 | 2.44E-04 | bZIP transcription factor = *Vitis vinifera* | |
|  | Q40172 | 0.00 | 25.05 | 1.22E-04 | MADS-box protein, TDR8 = *Solanum lycopersicum* | |
|  | D7UE94 | 0.00 | 16.29 | 1.22E-04 | Homeobox protein = *Vitis vinifera* | |
|  | Q7Y0Z5 | 0.00 | 11.83 | 2.44E-04 | Homeobox protein, Knotted homeodomain protein 4, KN4 = *Solanum lycopersicum* | |
|  | Q6RJ36 | 0.00 | 14.10 | 4.88E-04 | AP2/ERF, ERF2 = *Solanum lycopersicum* | |
|  | Q00LP7 | 0.00 | 6.77 | 4.88E-04 | GRAS1 = *Solanum lycopersicum* | |
|  | Q00LP5 | 0.00 | 5.01 | 9.77E-04 | GRAS4 = *Solanum lycopersicum* | |
|  | B9RI29 | 0.00 | 13.38 | 2.44E-04 | NAC protein = *Ricinus communis* | |
|  | Q6V398 | 0.00 | 5.16 | 9.77E-04 | EIN3/EIL = *Petunia hybrida* | |
|  | B9SU43 | 0.00 | 17.47 | 1.22E-04 | Heat shock factor protein, HSF = *Ricinus communis* | |
|  | B9T0T3 | 0.00 | 6.70 | 9.77E-04 | Zinc finger protein = *Ricinus communis* | |
|  | A6NAB4 | 0.00 | 4.93 | 1.95E-03 | bHLH transcription factor, MYC2 = *Vitis vinifera* | |
|  | B9STH6 | 0.00 | 13.70 | 1.95E-03 | bHLH transcription factor = *Ricinus communis* | |
|  | A5C8Z8 | 0.00 | 6.63 | 3.91E-03 | bHLH transcription factor = *Vitis vinifera* | |
|  | B9R797 | 0.00 | 7.48 | 7.81E-03 | bHLH transcription factor = *Ricinus communis* | |
|  | B9R7B6 | 0.00 | 7.11 | 1.95E-03 | bZIP transcription factor = *Ricinus communis* | |
|  | D7TX81 | 0.00 | 4.98 | 7.81E-03 | bZIP transcription factor = *Vitis vinifera* | |
|  | D9ZIR0 | 0.00 | 6.70 | 7.81E-03 | bZIP transcription factor, bZIP21 = *Malus domestica* | |
|  | D9ZIR3 | 0.00 | 14.96 | 7.81E-03 | bZIP transcription factor, bZIP4 bZIP40 = *Malus domestica* | |
|  | Q9SM50 | 0.00 | 14.77 | 7.81E-03 | bZIP transcription factor, LeHY5 = *Solanum lycopersicum* | |
|  | O65135 | 0.00 | 14.78 | 1.95E-03 | MADS-box protein, APETALA3, LeAP3 = *Solanum lycopersicum* | |
|  | E0CSD7 | 0.00 | 7.16 | 1.95E-03 | Homeobox protein = *Vitis vinifera* | |
|  | B9RNZ6 | 0.00 | 5.85 | 3.91E-03 | Homeobox protein knotted-1 = *Ricinus communis* | |
|  | D7SV02 | 0.00 | 12.18 | 3.91E-03 | Homeobox protein = *Vitis vinifera* | |
|  | D7TH52 | 0.00 | 5.72 | 3.91E-03 | E2F = *Vitis vinifera* | |
|  | D9ZJF0 | 0.00 | 8.08 | 3.91E-03 | WRKY transcription factor 11, WRKY11 = *Malus domestica* | |
|  | O22174 | 0.00 | 17.05 | 1.95E-03 | AP2/ERF, ERF008, At2g23340 | |
|  | D5L105 | 0.00 | 14.90 | 3.91E-03 | AP2/ERF, AP2D21 = *Malus domestica* | |
|  | D9ZJ69 | 0.00 | 8.50 | 1.95E-03 | MYB93 = *Malus domestica* | |
|  | A5BNN5 | 0.00 | 0.45 | 7.81E-03 | Zinc finger protein = *Vitis vinifera* | |
|  | B9RYR1 | 0.00 | 7.62 | 7.81E-03 | Zinc finger protein = *Ricinus communis* | |
